# Understanding the Impact of Drought on Foliar and Xylem Invading Bacterial Pathogen Stress in Chickpea

**DOI:** 10.3389/fpls.2016.00902

**Published:** 2016-06-21

**Authors:** Ranjita Sinha, Aarti Gupta, Muthappa Senthil-Kumar

**Affiliations:** National Institute of Plant Genome ResearchNew Delhi, India

**Keywords:** chickpea, combined stress, biotic-abiotic stress interaction, drought, *Ralstonia solanacearum*, *Pseudomonas syringae* pv. phaseolicola

## Abstract

In field conditions, plants are concurrently exposed to multiple stresses, where one stressor impacts the plant's response to another stressor, and the resultant net effect of these stresses differs from individual stress response. The present study investigated the effect of drought stress on interaction of chickpea with *Pseudomonas syringae* pv. phaseolicola (Psp; foliar pathogen) and *Ralstonia solanacearum* (Rs; xylem inhabiting wilt causing pathogen), respectively, and the net-effect of combined stress on chlorophyll content and cell death. Two type of stress treatments were used to study the influence of each stress factor during combined stress, viz., imposition of drought stress followed by pathogen challenge (DP), and pathogen inoculated plants imposed with drought in course of pathogen infection (PD). Drought stress was imposed at different levels with pathogen inoculum to understand the influence of different stress intensities on stress interaction and their net impact. Drought stressed chickpea plants challenged with Psp infection (DPsp) showed reduced *in planta* bacterial number compared to Psp infection alone. Similarly, Rs infection of chickpea plants showed reduced *in planta* bacterial number under severe drought stress. Combined drought and Psp (DPsp) infected plants showed decreased cell death compared to plants infected only with Psp but the extent of cell death was similar to drought stressed plants. Similarly, chlorophyll content in plants under combined stress was similar to the individual drought stressed plants; however, the chlorophyll content was more compared to pathogen only infected plants. Under combined drought and Rs infection (DRs), cell death was similar to individual drought stress but significantly less compared to only Rs infected plants. Altogether, the study proposes that both stress interaction and net effect of combined stress could be majorly influenced by first occurring stress, for example, drought stress in DP treatment. In addition, our results indicate that the outcome of the two stress interaction in plant depends on timing of stress occurrence and nature of infecting pathogen.

## Introduction

Chickpea (*Cicer arietinum*) is an important agricultural crop as well as second largest produced legume in the world (Gaur et al., 2012). However, the global productivity of chickpea is continually challenged by abiotic and biotic stresses. Chickpea plants are vulnerable to prolonged drought stress which causes around 40–50% yield loss (Gaur et al., 2012). In addition, biotic stresses including wilt (caused by *Fusarium oxysporum*) and foliar diseases such as Ascochyta blight (caused by *Ascochyta rabiei*) and botrytis gray mold (caused by *Botrytis cinerea*) have devastating effect on chickpea cultivation (Nene et al., 2012). In view of this, several studies have been pursued to understand the molecular mechanism of stress tolerance in response to individual stresses (Jha et al., 2014; Li et al., 2015); however, this knowledge could not be directly extrapolated for improving the stress tolerance against combined stresses. Plants in field conditions are continually exposed to multiple abiotic and biotic stresses, which results in altered physiological and biochemical changes and ultimately influence yield (Ramegowda and Senthil-Kumar, 2014; Suzuki et al., 2014), and therefore, investigating the impact of combined stress is imperative in plant stress biology. Studies indicate that the plants exhibit certain unique physiological and molecular responses in addition to several common responses for circumventing the combined effect of these stresses (Choi et al., 2013; Prasch and Sonnewald, 2013; Gupta et al., 2016b).

In combined stress scenario, drought can positively or negatively affect pathogen infection (Mattson and Haack, 1987). Drought stress may also influence the pathogen virulence or pathogenicity, resulting in upsurge of different potential pathogens not known earlier (Desprez-Loustau et al., 2007; Yáñez-López et al., 2012). Previous reports have shown that drought increases the susceptibility of plant to bacterial pathogens (Mohr and Cahill, 2003; Choi et al., 2013). In chickpea, drought stress has been shown to predispose the plant and significantly increase the incidence of dry root rot caused by *Rhizoctonia bataticola* (Sharma and Pande, 2013). Contrastingly, drought stress has also been shown to enhance the tolerance toward bacterial pathogen (Ramegowda et al., 2013; Gupta et al., 2016a). On the other hand, pathogens are also shown to influence plant-water relations (Mattson and Haack, 1987; Beattie, 2011). For example, pathogen can cause water soaking in infected leaf (Beattie, 2011) and vascular wilts can induce physiological drought stress on plants (Yadeta and Thomma, 2013). The two co-occurring stressors can modulate plant responses in a way different from when the two stressors occur independently. Earlier evidences suggest that the net effect of drought and bacterial pathogen combination on plant physiology and yield is different from the individual stresses. For example, *Xylella fastidiosa* (causal agent of Pierce's disease) infection in *Vitis vinifera* under drought stress showed increase in disease symptoms and decrease in leaf water potential, net CO_2_ assimilation, stomatal conductance, and transpiration rate (Choi et al., 2013).

The present study was conducted in chickpea plants exposed to combined drought stress and infection with *Pseudomonas syringae* pv. phaseolicola (Psp; foliar bacterial pathogen) and *Ralstonia solanacearum* (Rs; xylem inhabiting wilt causing bacterial pathogen) for testing three notions; (1) the impact of one stress on plant's interaction with other stress; (2) influence of order of stress occurrence and severity of each stress on the outcome of stress interaction; and (3) difference in the net impact of combined stress compared to two independent stresses.

Psp causes halo blight in broad bean (Saettler, 1991), a legume closely related to chickpea (Zhu et al., 2005). Halo blight appears as water soaked lesions. Rs is known to infect more than 200 plants species (Genin, 2010) including *Medicago truncatula*, another species closely related to chickpea and various other legume plants (Vailleau et al., 2007). Rs colonizes xylem tissue and secretes exopolysaccharides which inhibits the water supply of host plant which eventually results in vascular dysfunction and wilting (Genin, 2010).

## Materials and methods

### Plant material and growth conditions

Seeds of *Cicer arietinum* varieties PUSA 372 (procured from Indian Agriculture Research Institute, New Delhi) and ICC 4958 (available in our institute) were germinated in pots (3 inch in diameter) having a mixture of air dried peat (Prakruthi Agri Cocopeat Industries, Karnataka, India) and vermiculite (3:1, vol/vol) (Keltech Energies Pvt Ltd., Maharashtra, India) in an environmentally controlled growth chamber (PGR15, Conviron, Winnipeg, Canada) with diurnal cycle of 12-h-light/12-h-dark, 200 μE m^−2^s^−1^ photon flux intensity, 22°C temperature and 70% relative humidity. Pots were bottom irrigated every 2 days with half strength Hoagland's medium (**TS1094**, Hi-media Laboratories, Mumbai, India).

### Bacterial pathogen inoculum preparation

Pure culture of bacterial pathogens, viz. *Pseudomonas syringae* pv. phaseolicola (Psp) and *Ralstonia solanacearum* (Rs, procured from Indian type culture collection BI0001), IARI, New Delhi were used in this study. A single colony of Psp was inoculated in King's B (KB) medium (**M1544**, Hi-media Laboratories, Mumbai, India) supplemented with rifampicin (50 μg/mL) and incubated at 28°C with a continuous shaking of 200 rpm for 12 h. Rs was inoculated in LB medium (**M124**, Hi-media Laboratories, Mumbai, India) (without antibiotic) and incubated at 28°C with a continuous shaking of 200 rpm for 4 h. Both Psp and Rs were grown till the optical density (OD_600_) reached 0.6 and the cultures were pelleted down at 3500 g for 10 min. The pellets were washed twice with sterile distilled water and diluted to desired concentrations by re-suspending in sterile distilled water. The OD_600_ = 0.005 corresponding to 7 × 10^5^ colony forming units (cfu) /mL for Rs and 2.5 × 10^6^ cfu/mL for Psp were used for infecting the plants. Cfu corresponding to desired OD was calculated by plating the different dilutions for OD_600_ = 0.005.

### Pathogen inoculation

To study the pathogenicity of bacterial strains in chickpea ICC4958 (12-d-old), Psp suspension corresponding to 2.5 × 10^6^ cfu/mL was syringe infiltrated into the leaves and *in planta* bacterial number was determined from 0 to 10 days post-inoculation (dpi). Rs suspension (7 × 10^5^ cfu/mL) was vacuum infiltrated into the plants. For this, plants were placed inverted in a beaker containing Rs suspension with 0.02% Silwet L77 (Lehle seeds, Fisher Scientific, MA, USA) and vacuum of 8.7 psi was applied for 10 min. Plants were rinsed in water immediately after infiltration and *in planta* Rs number and phenotypic symptoms were recorded from 0 to 10 dpi. The leaf infiltration of Rs was previously reported in tobacco leaves (Kiba et al., 2003), and the infected plants displayed phenotypic disease symptoms similar to the symptoms observed by root inoculation method (Kanda et al., 2003; Shinohara et al., 2005). Similarly, syringe infiltration technique used for inoculation of Psp is a well-established technique (Liu et al., 2015).

### Drought imposition

Chickpea plants were grown in a pre-weighed pot mix and were subjected to drought stress by withholding the water supply. Drought stress levels were measured in terms of pot mix field capacity (FC) using gravimetric method (Reynolds, 1970), wherein for example, plants at FC 20% perceived 80% drought. Three drought levels viz. 60, 40, and 20% FC were used in the study and they were termed as mild, moderate and severe drought, respectively (Supplementary Table S1). A pot mix pre-maintained at 80% FC (with plant) took 2, 4, and 6 days to achieve 60, 40, and 20% FC, respectively, after withholding the water. In order to achieve all the drought levels on the same day, water was withheld on every alternate day for 3 batches, which resulted in generation of three sets of plants at 20, 40, and 60% FC on the sixth day. Plants with 80% FC were maintained as controls (Supplementary Figure S1). The respective FCs were maintained by adding the lost amount of water, till the end of the experiment.

### Combined stress imposition

Two methods were used for imposing combined stress, viz. drought followed by pathogen (DP) and pathogen followed by drought (PD). For DP studies, chickpea plants (20-d-old) with 60, 40, and 20% FC were vacuum infiltrated with Psp (OD_600_ = 0.005; 2.5 × 10^6^ cfu/mL) and Rs (OD_600_ = 0.005; 7 × 10^5^ cfu/mL) using aforementioned protocols. The pot surfaces were sealed with cellophane tape to avoid the entry of bacterial suspension into the pot mix (which may otherwise change the FC). After inoculation, the plants were sprayed with water and surface water was removed by blotting. Plants infiltrated with water (supplemented with 0.02% Silwet L77) were treated as mock.

For PD studies, chickpea plants were vacuum infiltrated with 2.5 × 10^6^ cfu/mL of Psp and 7 X 10^5^ cfu/mL of Rs following above mentioned protocol. Plants infiltrated with sterile water (supplemented with 0.02% Silwet L77) were treated as mock. Drought stress was imposed on plants 1 day after bacterial infection. A batch of plants infected with Psp and Rs was maintained without drought stress treatment (pathogen only stress, 80% FC). Similarly, a batch of uninfected plants subjected to drought stress only was maintained. Absolute control plants without bacterial as well as drought treatments were maintained at 80% FC. The experimental design for combined stress imposition is summarized in Supplementary Figures S1, S2.

### Sample harvest

Chickpea leaflets were harvested from the third twig (from hypocotyl). For DP stress, three leaflets from the same leaf were collected at 0 dpt for *in planta* bacterial multiplication assay. At 2 dpt, one leaf was collected for RNA isolation and 3 leaflets from leaf was used for *in-planta* bacterial multiplication. At 6 dpt, 3 leaflets for *in-planta* bacterial multiplication and 2 leaflet for cell death were collected from the same leaf. Three leaflet sample for the total chlorophyll and 3 leaf for phenotypic assessment were collected at 12 dpt. For PD stress, leaflet samples were collected at 0, 1, 2, 3, and 10 days post infection. The technical replicates were collected from same leaf. The methodology adopted for the sample collection and other experimental details are illustrated in detail in Supplementary Figures S1–S3.

### Assay for quantification of *in planta* bacterial number

The infected leaflets were surface sterilized with 0.01% H_2_O_2_ for 5 s, weighed and homogenized in 100 μL of sterile water. The homogenate was serially diluted in sterile water and the dilutions were plated on KB agar medium supplemented with rifampicin and on LB medium for assaying Psp and Rs counts, respectively. Total bacterial numbers were calculated as Log (cfu/mg fresh weight of leaf; Wang et al., 2012) and Log (cfu/mg dry weight of leaf) (Supplementary Figure S9).

Bacterial number (Cfu/mg) was calculated using the formula:

$$\begin{array}{l} \text{Cfu}∕\text{mg}=\frac{\frac{\text{Number of colonies }\times \text{ volume of homogenate }\times \text{ dilution factor}}{\text{volume plated}}}{\text{weight of the leaflet }\left(\text{mg}\right)} \end{array}$$

### Estimation of total chlorophyll content

Chlorophyll content of chickpea leaf discs [12.57 mm^2^ (4 mm diameter)] was determined at 12 dpt for DP, drought only, pathogen only, absolute control and mock control samples using method described by Hiscox and Israelstam (1980) with minor modifications. Leaves were incubated in 1 mL of dimethyl sulfoxide (DMSO): acetone (1:1 vol/vol) mix at room temperature in dark condition for 72 h for total chlorophyll extraction. Absorbance of extracts was read using Shimadzu UV 1800 spectrophotometer (Shimadzu Corporation, Kyoto, Japan) at 645 and 663 nm. Total chlorophyll content was calculated according to Arnon's equation (Arnon, 1949).

$$\begin{array}{l} \text{Total chlorophyll content }\left(\text{μg}∕{\text{cm}}^{2}\right)\\ =\frac{\left[\left(\text{ml solvent}\right)\left(20.2\;\times \text{ absorbance }645\right)\;+\;\left(8.02\;\times \text{ absorbance }663\right)\right]}{\text{leaf area }\left(\text{mm square}\right)} \end{array}$$

### Cell death

Cell death assay was performed as described by Koch and Slusarenko (1990) with minor modifications. Leaf samples from DP, drought only, pathogen only, absolute control and mock control were immersed in lactophenol-trypan blue for 12 h at room temperature followed by overnight de-staining in chloral hydrate (500 gm dissolved in 200 mL water). Lactophenol-trypan blue was prepared by dissolving 10 mL of lactic acid, 10 mL of glycerol, 10 g of phenol and 10 mg of trypan blue in 10 mL of distilled water. Cell death was observed under bright field microscope (Nikon Eclipse 80i, Nikon Corporation, Tokyo, Japan). The intensity of trypan blue staining was quantified using ImageJ software (http://imagej.nih.gov/ij/) (Schneider et al., 2012).

### Real-time PCR analysis

Expression profiles of genes responsive to drought (*CaLEA1, CaLEA2, CaLEA4, CaDREB2A*, and *CaNCED1*) and pathogen (*CaPAL2* and *CaPR4*) in DP (2 dpt), PD (10 dpt) and their respective individual stressed samples were analyzed by quantitative real-time PCR (RT-qPCR) (gene list with accession number given in Supplementary Table S2). Total RNA from leaf samples (100 mg fresh weight) was isolated using TriZol reagent (Cat # **15596018**, Thermo Fisher Scientific, California, USA) following manufacturer's guidelines. RNA quality was ascertained by agarose gel electrophoresis and quantified using NanoDrop ND-1000 spectrophotometer (Thermo Scientific, MA, USA). RNA samples with OD ratios in the range of 1.9–2.1 at 260/280 nm, and 2.0–2.3 at 260/230 nm were used for cDNA synthesis. First strand cDNA was synthesized using Verso cDNA synthesis kit (Cat # **K1621**, Thermo Fisher Scientific, MA, USA) from 5 μg of DNase treated total RNA in a reaction volume of 50 μL. The primers used in this study were synthesized from Sigma-aldrich, USA (Supplementary Table S2). Reaction mix comprised of 1 μL of 5 fold-diluted cDNA, 1 μL of each primer (10 μM/μL) and 5 μL of SYBR Green PCR master mix (Cat # **4309155**, Thermo Fisher Scientific, MA, USA) in a final volume of 10 μL. The reaction was run in ABI Prism 7000 sequence detection system (Applied Biosystems, California, USA). *CaACT1* (EU529707.1) and *Ca18S* (AJ577394.1) genes were used as endogenous control, and the cycle threshold (Ct) values obtained for these genes were used to normalize the data for PD and DP experiments, respectively. Relative fold change in gene expression was quantified using 2^−Δ*ΔCt*^ method (Livak and Schmittgen, 2001). Expression analysis was carried out using two independent biological replicates. For statistical analysis, the relative quantification value (RQ) was transformed to log_2_ value and test of significance was performed by one sample *t*-test. Relative transcript abundance of the chosen genes in DP, PD, and pathogen only samples was normalized with mock control, and expression profile of these genes in drought only sample was compared with absolute control.

### Statistical analysis

Data represented in the present study is derived from single experiment. Number of replicates for each experiment is mentioned in figure legends. Data is presented as the mean of replicates and error bars represent standard error of the mean. Number of replicates used in different experiments are also mentioned in Supplementary Figure S3. Test of significance used are one-way ANOVA, two-way ANOVA followed by *post-hoc* Tukey's test (*p* < 0.05), Student's *t*-test and one sample *t*-test. All the statistical analysis was done using SigmaPlot 11.0 (Systat Software, Inc).

## Results

### Assessment of bacterial pathogenicity and combined stress imposition

**Figure 1:**
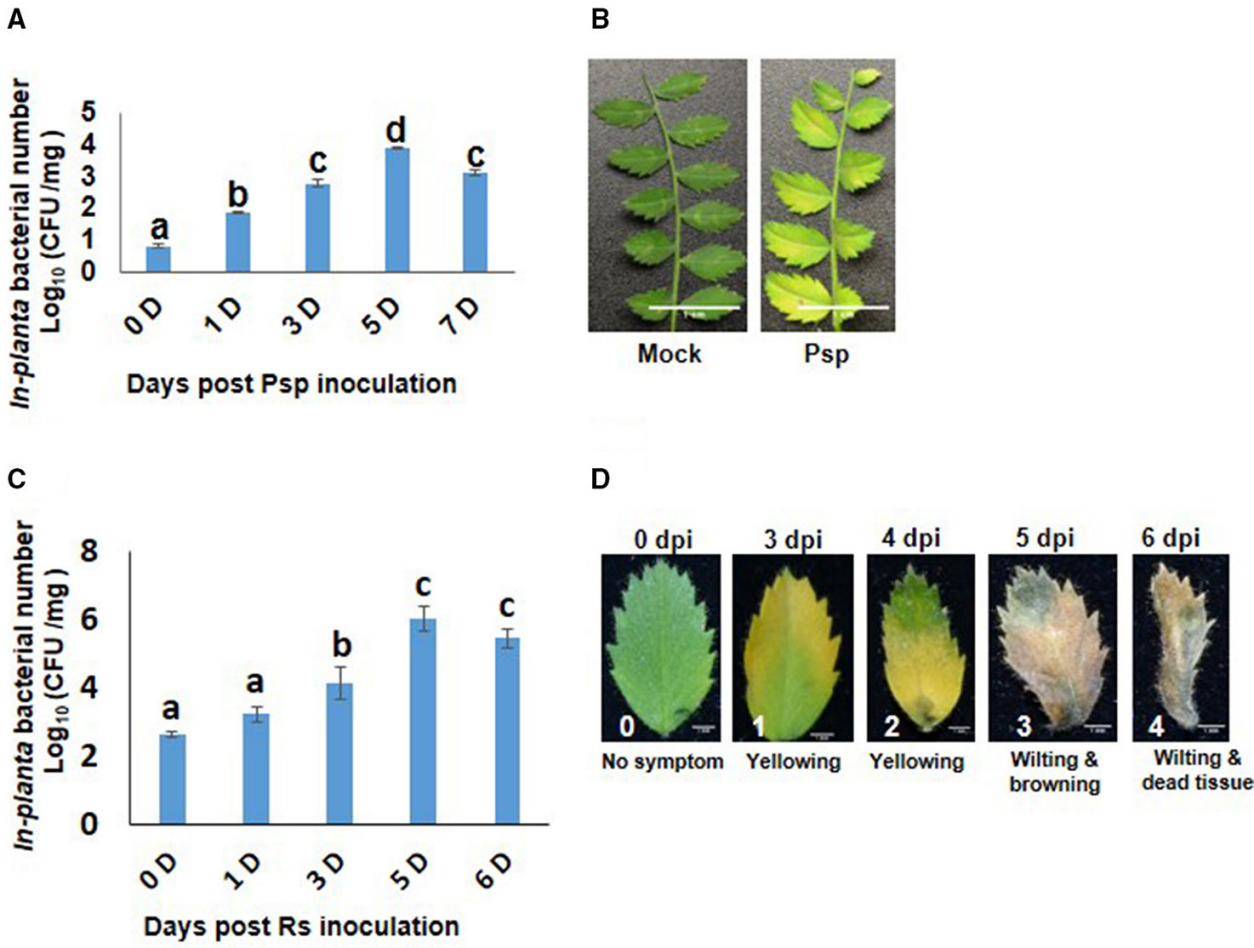
***Pseudomonas syringae* pv. phaseolicola (Psp) and *Ralstonia solanacearum* (Rs) multiplication and disease symptoms in chickpea**. Pathogenicity of Psp and Rs in chickpea ICC4958 was assessed by *in planta* bacterial multiplication assay in plants syringe inoculated with 7 × 10^5^ cfu/ml and 2.5 × 10^6^ cfu/ml of bacterial inoculum respectively. The multiplication was studied from 0 to 7 days post inoculation. Data is represented as Log (cfu/mg) and average of 2 replicates and ± SEM as error bar is plotted for Psp in graph **(A)** and for Rs in graph **(C)** is average of 5 replicate. Different letters above bar represents significant difference between means. One-way ANOVA was used for test of significance (*p* < 0.05). Disease symptoms in leaves were observed at 7 days post inoculation in Psp **(B)**. Disease symptoms for Rs was observed from 3 days post infection. The disease symptoms varied from yellowing to cell death to wilting with increase in *in planta* Rs count **(D)**.

Chickpea plants infected with Psp and Rs were initially assessed for their pathogenicity by determining *in planta* bacterial number and disease symptom development. Psp inoculated chickpea leaves showed increase in bacterial number till 5 dpi (Figure 1A) and chlorosis was observed on the inoculated leaves at 6 dpi (Figure 1B). These results indicated that Psp is a potential but mild pathogen of chickpea. Rs infiltrated plants showed increase in number of bacterial colony forming units till 5 dpi (Figure 1C), and this was accompanied with appearance of disease symptoms such as yellowing at low bacterial numbers [4.86 Log (cfu/mg)], and wilting and cell death at higher bacterial numbers [6.54 Log (cfu/mg)]. This demonstrated that Rs is also a potential host pathogen of chickpea (Figure 1D).

The effect of combined stress was studied using two methods, viz. (i) drought stress followed by pathogen infection (DP), and (ii) pathogen infection followed by drought stress imposition (PD) as detailed in “methods” section (Supplementary Figures S1–S3, Supplementary Table S2).

### Combined stress reduced multiplication of Psp and decreased cell death along with increased chlorophyll content compared to infection with pathogen alone

**Figure 2:**
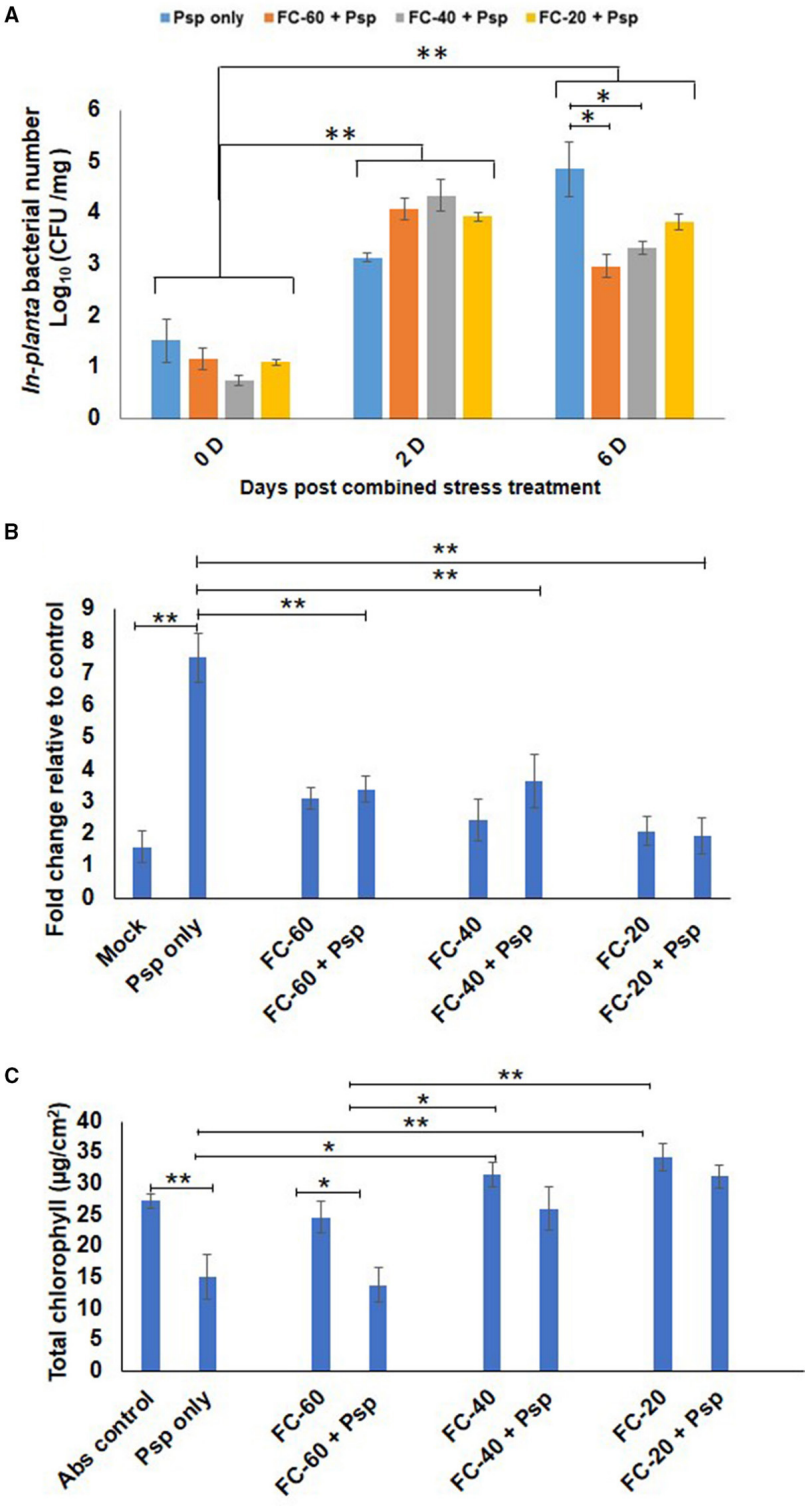
**Effect of different levels of drought stress on *P. syringae* pv. phaseolicola multiplication and the net effect of combined stress on chickpea**. *In-planta* number of Psp was measured for the combined stresses plants (mild, moderate, and severe DPsp) and only Psp stress. It was measured for 0, 2, and 6 days post combined stress treatment (dpt). Data is represented as Log (cfu/mg) in graph **(A)**. Each bar is average of 9 replicates and error bar represents ± SEM. Cell death was studied using trypan blue staining method. The sample was photographed under bright field microscope and intensity of trypan blue was measured using ImageJ software. Graph **(B)** represents quantitative measurement of cell death in drought and pathogen stressed samples at 6 dpt. Bar represents fold cell death over absolute control and error bar represents ± SEM, each bar is average of four replicates. Graph **(C)** represents total chlorophyll content of different combined stress and individual stresses measured at 12 dpt. Each bar represents average of 6 replicates and error bar represents ± SEM. ^*^, ^**^represents significant difference at *p* < 0.01 and *p* < 0.001, respectively. Two-way ANOVA was used for test of significance and *post-hoc* Tukey's test was used to represent significant difference between the means.

As a result of stress interaction, 1.6 and 1.5 fold significant decrease in the bacterial number of Psp was observed under mild and moderate drought stress, respectively, when compared to their number in plants challenged with Psp alone (Figure 2A). The net effect of combined drought and Psp (DPsp) in chickpea was further assessed by determining cell death and total chlorophyll content. In the present study, prominent cell death at 6 dpt was observed in plants individually challenged with both stresses (Figure 2B, Supplementary Figure S4A). A three-fold increase in cell death compared to control sample was observed in leaves of mild drought stress. However, there was no significant change in the extent of cell death observed in response to increase in drought severity. Both moderate and severe drought stressed plants exhibited approximately a two-fold increase in cell death in comparison to control plants (Figure 2B). Psp infection lead to 4.68- and 7-fold increase in cell death compared to mock and absolute control, respectively. Decrease in cell death was observed in DPsp stressed plant when compared to plants infected with Psp alone. However, extend of cell death was similar in both DPsp and drought stressed plants (Figure 2B). The mild DPsp (mild drought with Psp infection), moderate DPsp (moderate drought with Psp infection) and severe DPsp (severe drought with Psp infection) showed 2.2-, 2.05-, and 3.8-fold decrease in cell death, respectively, in comparison to plants challenged with Psp infection alone (Figure 2B).

In case of total chlorophyll content, a significant 1.8 fold decrease was observed in Psp infected leaves than control leaves. In contrary, plants exposed to only drought and DPsp stresses did not show any change in their chlorophyll content while compared to control plants (Figure 2C). However, the chlorophyll content was significantly higher in DPsp stressed plants than plants infected with Psp alone. There was 1.7- and 2-fold more chlorophyll in moderate DPsp and severe DPsp stressed plants, respectively, when compared to plants infected with pathogen alone. Chlorophyll content in moderate and severe DPsp was unchanged in comparison to moderate and severe levels of drought stress alone, respectively. However, mild DPsp showed around 2-fold decrease in chlorophyll content over severe drought alone (Figure 2C). The phenotype recorded after 12 dpt showed chlorotic symptoms with disease score of 3.5, 2.3, and 1.6 for Psp alone, mild and moderate DPsp, respectively. Phenotype of severe DPsp was similar to mock control (Supplementary Figures S4B,C). Taken together, the net effect due to combined stress (DP) was similar to drought stress.

### Combined drought and Rs stress showed less Rs multiplication and cell death and more chlorophyll content over Rs only stress

**Figure 3:**
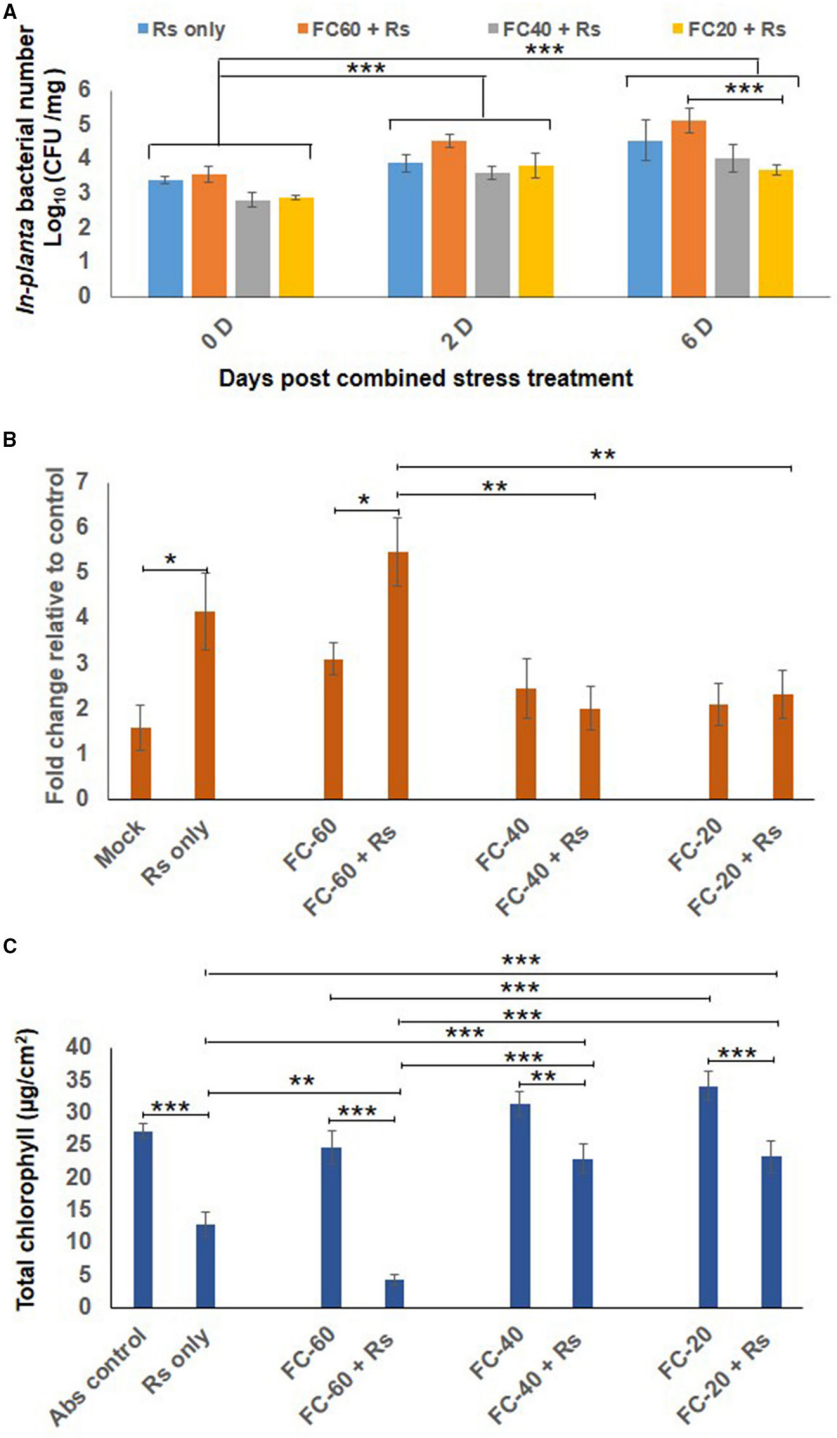
**Effect of drought stress on *in planta* multiplication of *R. solanacearum* (Rs) and net effect of combined stress on chickpea. (A)** The *in planta* bacterial number of Rs under combined stress (mild, moderate and severe DRs) and only Rs stress condition was measured at 0, 2, and 6 days after combined stress treatment (dpt). Data is represented as Log (cfu/mg) in graph **(A)**. Each bar is average of 9 replicates and error bar represents ± SEM. Cell death was studied by trypan blue staining method for combined stresses (DRs) as well as individual Rs and drought stresses. The samples were photographed under bright field microscope and intensity of trypan blue was measured using ImageJ software. Graph **(B)** shows average of four ImageJ intensity value. Y-axis represents fold change over control & error bar signifies ± SEM. Graph **(C)** shows total chlorophyll content in leaf disc of chickpea imposed with drought stresses, Rs infection and combined DRs. Chlorophyll estimation was done at 12 dpt. Each bar represents average of six replicates and error bar represents ± SEM. ^*^, ^**^, and ^***^ represents significant difference at *p* < 0.05, *p* < 0.01, and *p* < 0.001, respectively. Two-way ANOVA was used as test of significance and *post-hoc* Tukey's test was used to calculate significant difference between each mean.

The total bacterial count of Rs was constant in chickpea plants exposed to mild drought stress, but their number declined during severe drought stress as compared to plants infected with Rs only (at 6 dpt; Figure 3A). Similarly, cell death and total chlorophyll content were also assessed in combined stress treated plants as well as their respective controls. Increased cell death was observed in plants individually challenged with drought and Rs infection (Figure 3B, Supplementary Figure S5A). When compared to absolute control, drought stress caused more cell death by 3-, 2.4-, and 2-folds in mild, moderate and severe drought levels, respectively. Rs infection showed 4.15-fold increase in cell death compared to absolute control. During combined stress conditions, increase in cell death was noted in mild DRs (mild drought with Rs) plants compared to absolute control. However, moderate DRs (moderate drought with Rs) and severe DRs (severe drought with Rs) did not have a major impact on the viability of cells as they showed 2-and 1.7-fold reduction in cell death, respectively, compared to Rs alone stress (Figure 3B). Moreover, the extent of cell death observed during moderate and severe DRs, and drought stress alone were similar (Figure 3B).

Total chlorophyll content in the leaves of plants infected with pathogen alone (Rs) was reduced to 2-folds in comparison to control, and it further decreased in mild DR treatment (6-fold reduction compare to control; Figure 3C). There was 1.4-fold decrease in the chlorophyll content of plants challenged with moderate and severe DRs compared to plants at respective drought stress levels. However, the chlorophyll levels in mild and moderate DRs were 1.8-fold high contrasting to plants infected with Rs alone (Figure 3C). The difference in chlorophyll content was reflected on the phenotype of plants, as the plants challenged with both mild drought and Rs infection showed increased chlorosis in comparison to plants infected with Rs alone (Supplementary Figure S5C). Chlorotic symptoms with disease scores of 1.6, 4.3, and 1.6 for Rs alone and mild and moderate DRs respectively were recorded at 12 dpt. However, severe DPsp had phenotype similar to mock control with no chlorosis (Supplementary Figures S5B,C). Thus, with mild drought stress, the disease severity was decreased in case of DPsp, but was significantly increased in DRs combined stress.

These results suggest that the net effect of combined stress was more due to the drought stress and also, two pathogens differentially elicited net-effects on plants during combined stress as measured by cell death and chlorophyll content (Supplementary Figures S6A,B). Additionally, our results also indicate that level of drought stress decides elicitation or suppression of plant defenses (Supplementary Figure S6C).

### Bacterial multiplication was similar in plants challenged with PD combined stress and pathogen stress

**Figure 4:**
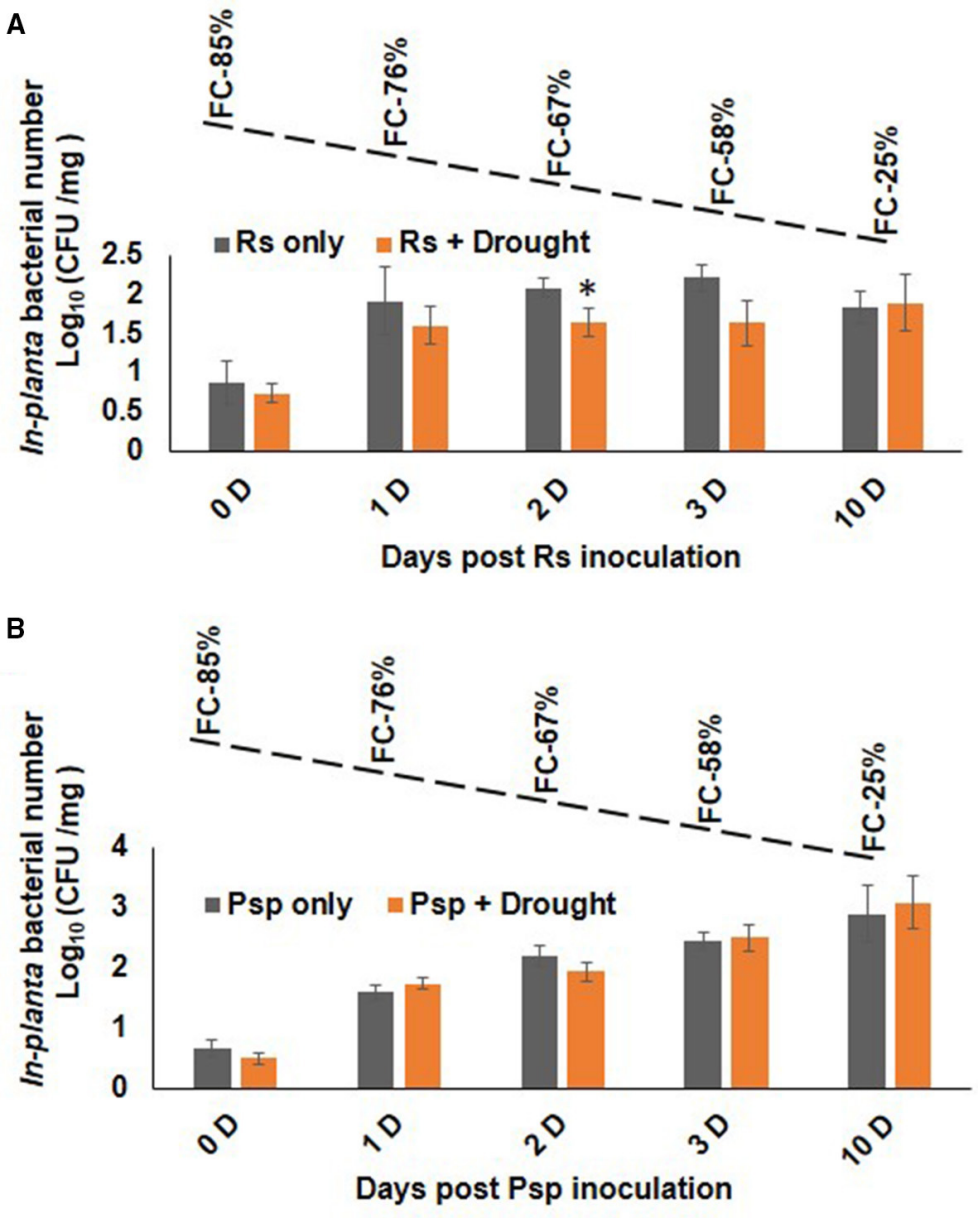
**Effect of drought stress on bacterial multiplication under PD combined stress**. The *in planta* presence of *R. solanacearum* and *P. syringae* pv. phaseolicola count was measured at 0, 1, 2, 3, and 10 days post infection under RsD (Rs+Drought), only Rs, PspD (Psp+Drought), and only Psp stress. Graph **(A)** represents the *in planta* count of Rs and graph **(B)** represents the *in planta* count of Psp. Each bar represents average of 9 biological replicates and error bar represents ± SEM. ^*^Represents significant difference at *p* < 0.05. Student's *t*-test was used for test of significance. Dotted line denotes gradual decline in soil moisture content with values representing potted plants at actual field capacity (FC) on respective days.

Plants infected with pathogen followed by imposition of drought stress (PD) showed similar bacterial number as that of pathogen only treatment (Figure 4). During progressive drought in PD stress, plants at 4 days post infection (dpi) experienced 60% FC and at 10 days post infection 20% FC (Supplementary Figure S2B). Therefore, PD stressed plant at 4 and 10 dpi experienced mild and severe combined stress respectively. Combined PD stressed (Psp) and Psp only infected plants showed a constant increase in bacterial count till 10 dpi. In contrary, Rs count increased significantly on 1 dpi in Rs only, but no notable increase was observed on subsequent days (Figure 4). However, PspD and RsD did not show significant decrease in bacterial count at mild or severe drought stress. Altogether, the dissimilar effect of DP and PD on bacterial colony number indicates that timing of occurrence of drought stress is important during stress interaction.

### Expression of pathogen stress responsive genes were differentially regulated under combined stress in comparison to individual stress

**Figure 5:**
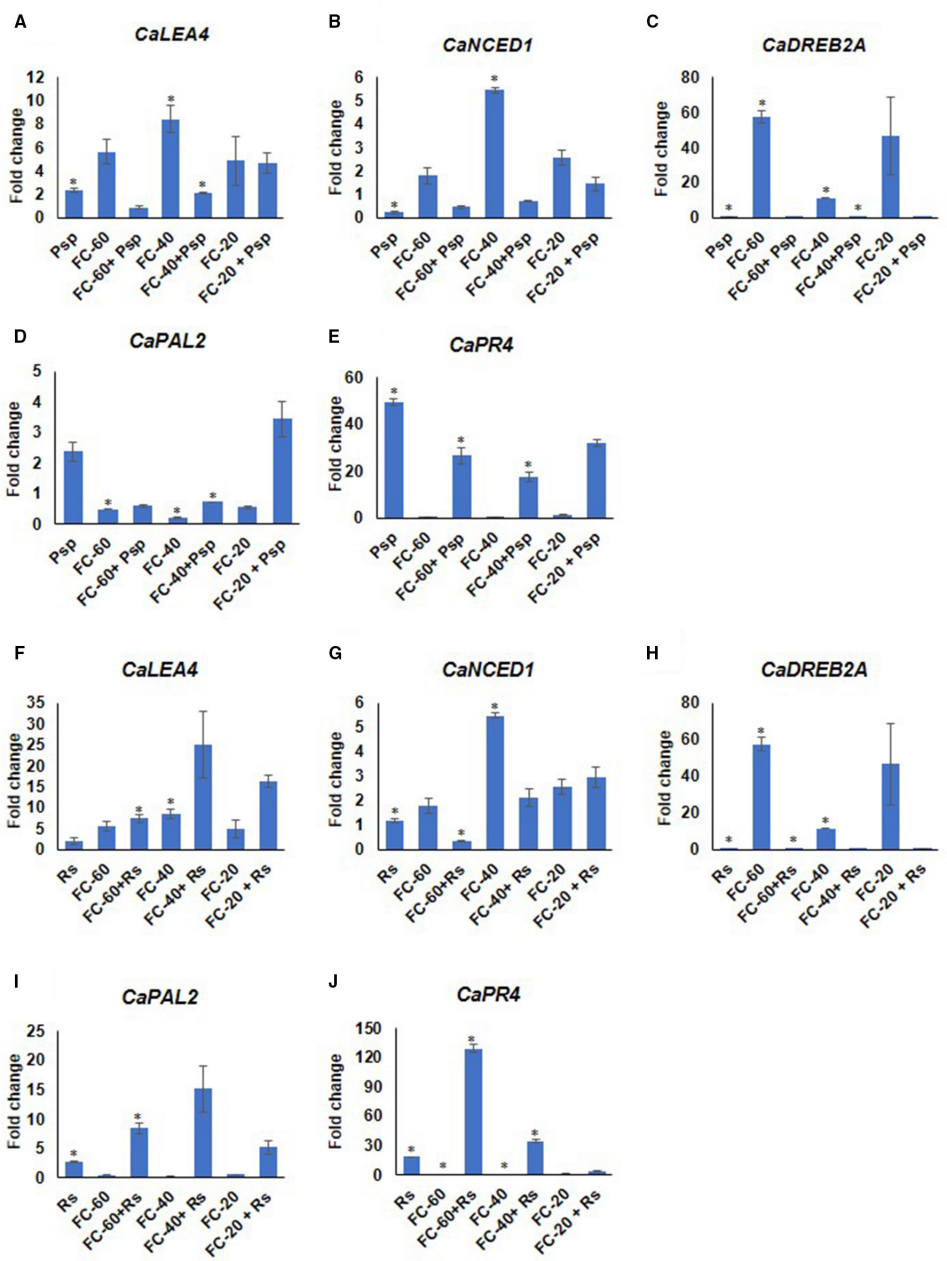
**Expression analysis of stress responsive genes of chickpea under drought, pathogen and combined stress (DP)**. Expression of stress responsive genes in comparison to control was studied using RT-qPCR. The Ct values of different genes were normalized with *Ca18S* internal control. Fold change in gene expression was calculated by 2^−ΔΔCT^ method. Mock was considered as reference for DP and pathogen only, and absolute control was reference for drought only. The differential gene expression for *CaLEA4* **(A,F)** *CaNCED1* **(B,G)** *CaDREB2A* **(C,H)** and *CaPAL2* **(D,I)** and *CaPR4* **(E,J)** under DPsp and DRs combined stress are represented as bar graph. Each bar signifies average of two biological replicates and error bar represents ± SEM. Significance was tested by one sample *t*-test. ^*^Denotes significant at *p* < 0.05.

Differential expression pattern of drought responsive (*CaNCED1*, 9-cis-epoxycarotenoid dioxygenase; *CaDREB2A*, dehydration responsive element binding; *CaLEA4*, late embryogenesis abundant 4) as well as pathogen responsive (*CaPR4*, thaumatin-like pathogenesis-related protein 4-like; *CaPAL2* phenylalanine ammonia-lyase 2-like) genes were observed during these stress conditions compared to corresponding mock and absolute control at 2 dpt (Figure 5). This further validated the stress experienced by the plants. The analysis showed higher expression of drought responsive genes in all the levels of drought stress. Among these genes, *CaDREB2A* showed 57.5 fold up-regulated expression during drought, whereas the expression of *CaLEA4* and *CaNCED1* increased from 5.6 and 1.7 to 8.4 and 5.4 folds, respectively, with the increase in severity of drought stress from mild to moderate levels (Figure 5). The pathogen responsive genes, *CaPR4* and *CaPAL2* showed downregulation during drought stress. Interestingly, both drought and pathogen responsive genes had almost similar expression under mild and severe drought alone stress (Figure 5). In case of pathogen challenge, *CaPR4* and *CaPAL2* displayed significant up-regulation in response to both the pathogens; however, *CaPR4* exhibited a relatively higher expression in response to Psp (49.4 fold) than Rs (18.4 fold) (Figure 5). Moreover, *CaLEA4* exhibited up-regulated expression pattern in pathogen alone (Psp, Rs) infected plants (Figure 5). While in response to DP combined stress, drought responsive genes, *CaDREB2A* and *CaNCED1* showed decreased expression compared to drought alone stress and decreased or similar expression compared to pathogen alone during mild and moderate in both DPsp and DRs stresses. However, *CaLEA4* showed decreased expression in DPsp but increased expression in DRs compared to both the individual stresses. The expression of pathogen stress responsive genes *CaPR4* and *CaPAL2* was downregulated in DPsp compare to Psp alone but they were up-regulated in combined DRs compared to individual stresses (Figure 5). Expression of *CaDREB2A, CaPR4*, and *CaPAL2* under severe DPsp and DRs was almost similar to respective pathogen alone stress. However, *CaLEA4* and *CaNCED1* showed slightly increased expression compare to pathogen alone.

**Figure 6:**
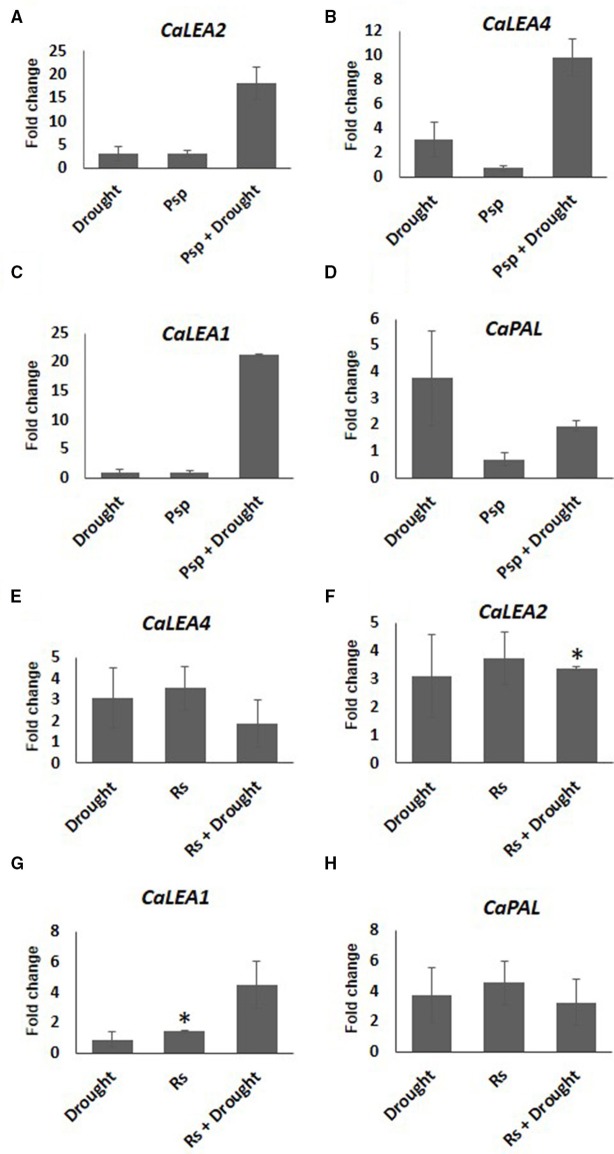
**Expression analysis of stress responsive genes of chickpea under drought, pathogen and combined stress (PD)**. Expression of stress responsive genes in comparison to control was studied using RT-qPCR. The Ct values of different genes were normalized with *CaActin* internal control. Fold change in gene expression was calculated by 2^−ΔΔCT^ method. Mock was considered as reference for PD and pathogen only and absolute control was reference for drought only. The differential gene expression for *CaLEA2* **(A,F)** *CaLEA4* **(B,E)** *CaLEA1* **(C,G)** and *CaPAL2* **(D,H)** under PspD and RsD combined stress are represented as bar graph. Each bar represents average of two biological replicates and error bar represents ± SEM. Significance was verified by one sample *t*-test. ^*^Denotes significant at *p* < 0.05.

Expression pattern of stress responsive genes was also studied in the samples where pathogen infection and subsequent drought stress imposition have been performed (PD). Increased expression of *CaLEA2* in response to drought alone, and *CaLEA4* and *CaPAL2* genes in pathogen alone at 10 days post-inoculation of pathogen confirmed the prevalence of drought and pathogen stress in individual stressed plants. Compared to mock control, *CaPAL2* showed lower expression in samples infected with Psp alone, however a four-fold higher expression of this gene was observed in response to infection with Rs alone (Figure 6). During combined PspD stress, *CaLEA4, CaLEA1*, and *CaLEA2* genes showed higher transcript expression compared to individual drought and pathogen stresses. However, during combined RsD stress, only *CaLEA1* showed 4.5 fold increased expression in comparison to individual stresses. This indicates that the alteration in stress responsive genes was influenced by the nature of infecting pathogen during combined stress response.

## Discussion

Simultaneous occurrence of drought and bacterial pathogen infection influences the impact of each other during their interaction *in planta* (Timmusk and Wagner, 1999; McElrone et al., 2003; Mohr and Cahill, 2003; Ramegowda et al., 2013). Moreover, the net impact of combined stress on plants has been reported to be unique compared to individual stresses (Choi et al., 2013). The present study tested the effect of drought stress on the pathogenicity of two different bacterial pathogens in chickpea, and also the net physiological effect by assessing cell death and chlorophyll content during stress combinations (DP and PD).

Our results indicated that pathogen infection preceded by drought stress reduced the multiplication of Psp and Rs in chickpea. There could be three possible reasons for this effect. First, presumably reduced availability of water needed for *in planta* bacterial multiplication. Beattie (2011) has explained that water influences plant-pathogen interaction. Earlier, reduced water potential in bacterial culture media was found to delay the bacterial multiplication (Beattie, 2011). It has also been shown that plants produce localized desiccation at the site of infection and reduced pathogen numbers as a part of basal and effector triggered defenses (Beattie, 2011). In our study, we found reduced leaf RWC in response to drought stress and combined stress (Supplementary Figure S7). Secondly, the drought stress induced molecular and biochemical adaptation in chickpea plants could have contributed to reduced bacterial growth. For example, drought stress provokes the accumulation of reactive oxygen species (ROS) which at lower concentrations acts as secondary messenger in signal transduction and triggers defense response against pathogen (Lamb and Dixon, 1997). In current study, we observed increased ROS production with increase in drought stress level (Supplementary Figure S8) and therefore priming with drought mediated ROS can be one of the reason for decreased bacterial multiplication. Similarly, *PR5* (pathogenesis-related protein-5) and *PDF2*.1 (plant defensin 1.2) genes, which are known to be involved in pathogen defense (Glazebrook, 2001) are also found to be highly expressed under drought stress (Ramegowda et al., 2013). Boominathan et al. (2004) have shown that drought adaptation increases the expression of serine threonine protein kinases (STPK) which are also involved in pathogen defense (Dangl and Jones, 2001; Zhang et al., 2013). In our study, we found increased expression of *CaPR4* in drought stress alone, and therefore, we assume that it primed the plant for upcoming pathogen stress. Third reason for reduced multiplication could be, physiological, biochemical and molecular mechanism that are unique to combined stress (Pandey et al., 2015). In this study, *CaPAL2* and *CaPR4* showed very high expression under combined stress over individual stresses in DRs. Such fold change was noted to be more than the additive expression of these genes under two individual stresses which could be taken up as unique response by plants under combined stress. Therefore, we assume that this could be one of the reason for the decreased multiplication under combined stress.

During DRs, multiplication of Rs was found to be decreased at severe drought level. However, mild drought stress did not reduce the Rs multiplication. This indicates that intensity of drought stress plays important role in combined stress effect. During stress interaction, the net outcome of the stress response decides whether the plant is capable of circumventing the combined stress effect or not. In the present study, we found that the drought stress (individual) leads to increased cell death but does not affect chlorophyll content of the plant, whereas pathogen infection lead to increase in cell death and disease associated decrease in chlorophyll content. However, the impact of DPsp stress on cell death and chlorophyll was similar to drought stress and it was reduced in comparison to only Psp stress. Similarly, the net effect of DRs except mild DRs on cell death was almost similar to drought stress and reduced in comparison to only Rs stress. This indicated that drought stressed plants were able to defend themselves better against upcoming pathogen.

In conclusion, the study demonstrates that priming of drought stress reduces the multiplication of Psp and Rs pathogens *in planta*. The net effect of combined stress was not additive and drought has more impact during combined occurrence with pathogen. The study also shows that the outcome of combined stress is conditional and depends on which stress factor occurs first in the plant.

## Author contributions

MS conceived the idea and provided resources. MS, RS designed the experiments. RS, AG performed the experiments. RS analyzed the data under the guidance of MS. MS, RS wrote the paper. All authors have read and approved the final manuscript.

### Conflict of interest statement

The authors declare that the research was conducted in the absence of any commercial or financial relationships that could be construed as a potential conflict of interest.
