# Supplementary material for: Understanding the Impact of Drought on Foliar and Xylem Invading Bacterial Pathogen Stress in Chickpea
Source: Front Plant Sci. 2016 Jun 21;7:902. doi: 10.3389/fpls.2016.00902 (PMC4914590; doi:10.3389/fpls.2016.00902)
Supplement: Supplementary file 1 [file Table1.DOCX]

| **Labels Used** | **Combined Stress** | **Individual Stress** | **Drought before Pathogen** | **Pathogen before Drought** | **Drought** | **Psp** | **Rs** |
| --- | --- | --- | --- | --- | --- | --- | --- |
| DPsp | Yes | No | Yes | No | Yes | Yes | No |
| DRs | Yes | No | Yes | No | Yes | No | yes |
| PspD | Yes | No | No | Yes | Yes | Yes | No |
| RsD | Yes | No | No | Yes | Yes | No | Yes |
| Psp only | No | Yes | No | No | No | Yes | No |
| Rs only | No | Yes | No | No | No | No | Yes |
| Drought only | No | Yes | No | No | Yes | No | No |

**Table S1**: **Details of the labels and treatments used in this study**
