# Supplementary material for: Understanding the Impact of Drought on Foliar and Xylem Invading Bacterial Pathogen Stress in Chickpea
Source: Front Plant Sci. 2016 Jun 21;7:902. doi: 10.3389/fpls.2016.00902 (PMC4914590; doi:10.3389/fpls.2016.00902)
Supplement: Supplementary file 2 [file Table2.DOCX]

| **S. No.** | **Genes** | **Complete name** | **Accession no.** | **References** | **Primer sequences** |
| --- | --- | --- | --- | --- | --- |
| **Drought stress responsive** | | | | | |
| 1 | CaLEA4 | Late embryogenesis abundant 4 | GU247512.1 | Olvera-Carrillo et al., 2010 | **FP**: GCTCACACCCGATTGGAACT  **RP**: AGACTAAACTTTGTGCAGTCCTT |
| 2 | CaDREB | Dehydration responsive element binding protein | DQ321719.1 | Liu et al., 1998 | **FP**: AGCACATGTTAGTGAAAAGCCA  **RP**: CAAGGCGGGCGTTCAGTT |
| 3 | CaNCED | 9-cis-epoxycarotenoid dioxygenase | AB771415.1 | Iuchi et al., 2000 | **FP**: ACCCACGTGTCCAAATCTCC  **RP**: CGGCTACCGGTTCGTAATGT |
| 4 | CaLEA1 | Late embryogenesis abundant 1 | AJ224518.1 | Veeranagamallaiah et al., 2011 | **FP**: GTGAGACCATGGGCCGAAC  **RP**: TTGGGCTGTCTGACTGGT |
| 5 | CaLEA2 | Late embryogenesis abundant 2 | AJ224519.1 | Veeranagamallaiah et al., 2011 | **FP**: AGGTGCAACTGATGCTGTGA  **RP**: GCGTTGAATAAAAACCAAATTACGA |
| **Pathogen stress responsive** | | | | | |
| 6 | CaPAL | Phenylalanine ammonia-lyase 2-like | NM_001279177.1 | Huang et al., 2010 | **FP**: AGGTGAAACGCATGGTGGAA  **RP**: ATCGTGTCTCTTCGGCAAGT |
| 7 | CaPR4 | Thaumatin-like pathogenesis-related protein 4-like | NM_001279109.1 | Singh et al., 2013 | **FP**: TCAGCACAAGGAGCAAGGTT  **RP**: TCGCGTGGATTTAACTGCCT |
| **Internal control** | | | | | |
| 8 | CaActin1 | actin | EU529707.1 |  | **FP:**  GATGTCCAGAGGTCCTGTTCC  **RP:**  CAGTACCACTTGTTGTCTAAAACC |
| 9 | Ca18S | 18S rRNA gene | AJ577394.1 |  | **FP:** GCCCGCGACGTTGTGA  **RP:** CCTTGTTACGACTTCTCCTTCCTCTA |

**Table S2: List of genes for expression analysis**

**FP, forward primer; RP, reverse primer**

**References**

**Huang J, Gu M, Lai Z, Fan B, Shi K, Zhou Y-H, Yu J-Q, Chen Z** (2010) Functional Analysis of the Arabidopsis PAL Gene Family in Plant Growth, Development, and Response to Environmental Stress. Plant Physiol **153**: 1526–1538

**Iuchi S, Kobayashi M, Yamaguchi-Shinozaki K, Shinozaki K** (2000) A stress-inducible gene for 9-cis-epoxycarotenoid dioxygenase involved in abscisic acid biosynthesis under water stress in drought-tolerant cowpea. Plant Physiol **123**: 553–562

**Liu Q, Kasuga M, Sakuma Y, Abe H, Miura S, Yamaguchi-Shinozaki K, Shinozaki K** (1998) Two transcription factors, DREB1 and DREB2, with an EREBP/AP2 DNA binding domain separate two cellular signal transduction pathways in drought- and low-temperature-responsive gene expression, respectively, in Arabidopsis. Plant Cell **10**: 1391–1406

**Olvera-Carrillo Y, Campos F, Reyes JL, Garciarrubio A, Covarrubias A a** (2010) Functional analysis of the group 4 late embryogenesis abundant proteins reveals their relevance in the adaptive response during water deficit in Arabidopsis. Plant Physiol **154**: 373–390

**Singh NK, Kumar KRR, Kumar D, Shukla P, Kirti PB** (2013) Characterization of a pathogen induced thaumatin-like protein gene AdTLP from Arachis diogoi, a wild peanut. PLoS One **8**: 1–18

**Veeranagamallaiah G, Prasanthi J, Reddy KE, Pandurangaiah M, Babu OS, Sudhakar C** (2011) Group 1 and 2 LEA protein expression correlates with a decrease in water stress induced protein aggregation in horsegram during germination and seedling growth. J Plant Physiol **168**: 671–677
