# Supplementary material for: Understanding the Impact of Drought on Foliar and Xylem Invading Bacterial Pathogen Stress in Chickpea
Source: Front Plant Sci. 2016 Jun 21;7:902. doi: 10.3389/fpls.2016.00902 (PMC4914590; doi:10.3389/fpls.2016.00902)
Supplement: Supplementary file 3 [file Presentation1.PPTX]

## Slide 1
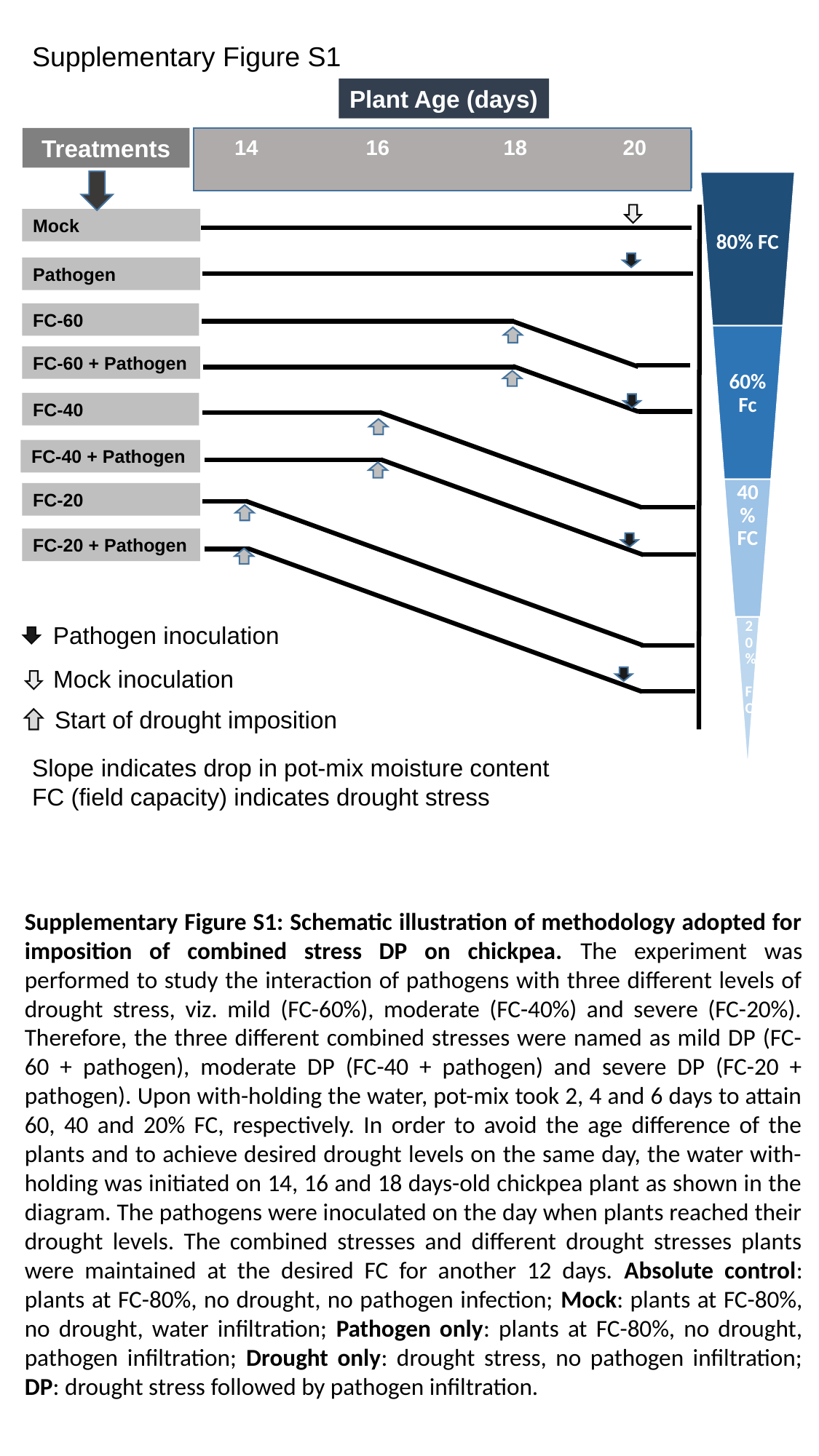

Supplementary Figure S1
Plant Age (days)
Treatments
 14 16 18 20
Mock
Pathogen
FC-60
FC-60 + Pathogen
FC-40
FC-40 + Pathogen
FC-20
FC-20 + Pathogen
Pathogen inoculation
Mock inoculation
Start of drought imposition
Slope indicates drop in pot-mix moisture content
FC (field capacity) indicates drought stress
Supplementary Figure S1: Schematic illustration of methodology adopted for imposition of combined stress DP on chickpea. The experiment was performed to study the interaction of pathogens with three different levels of drought stress, viz. mild (FC-60%), moderate (FC-40%) and severe (FC-20%). Therefore, the three different combined stresses were named as mild DP (FC-60 + pathogen), moderate DP (FC-40 + pathogen) and severe DP (FC-20 + pathogen). Upon with-holding the water, pot-mix took 2, 4 and 6 days to attain 60, 40 and 20% FC, respectively. In order to avoid the age difference of the plants and to achieve desired drought levels on the same day, the water with-holding was initiated on 14, 16 and 18 days-old chickpea plant as shown in the diagram. The pathogens were inoculated on the day when plants reached their drought levels. The combined stresses and different drought stresses plants were maintained at the desired FC for another 12 days. Absolute control: plants at FC-80%, no drought, no pathogen infection; Mock: plants at FC-80%, no drought, water infiltration; Pathogen only: plants at FC-80%, no drought, pathogen infiltration; Drought only: drought stress, no pathogen infiltration; DP: drought stress followed by pathogen infiltration.
