# Supplementary material for: Understanding the Impact of Drought on Foliar and Xylem Invading Bacterial Pathogen Stress in Chickpea
Source: Front Plant Sci. 2016 Jun 21;7:902. doi: 10.3389/fpls.2016.00902 (PMC4914590; doi:10.3389/fpls.2016.00902)
Supplement: Supplementary file 4 [file Presentation2.PPTX]

## Slide 1
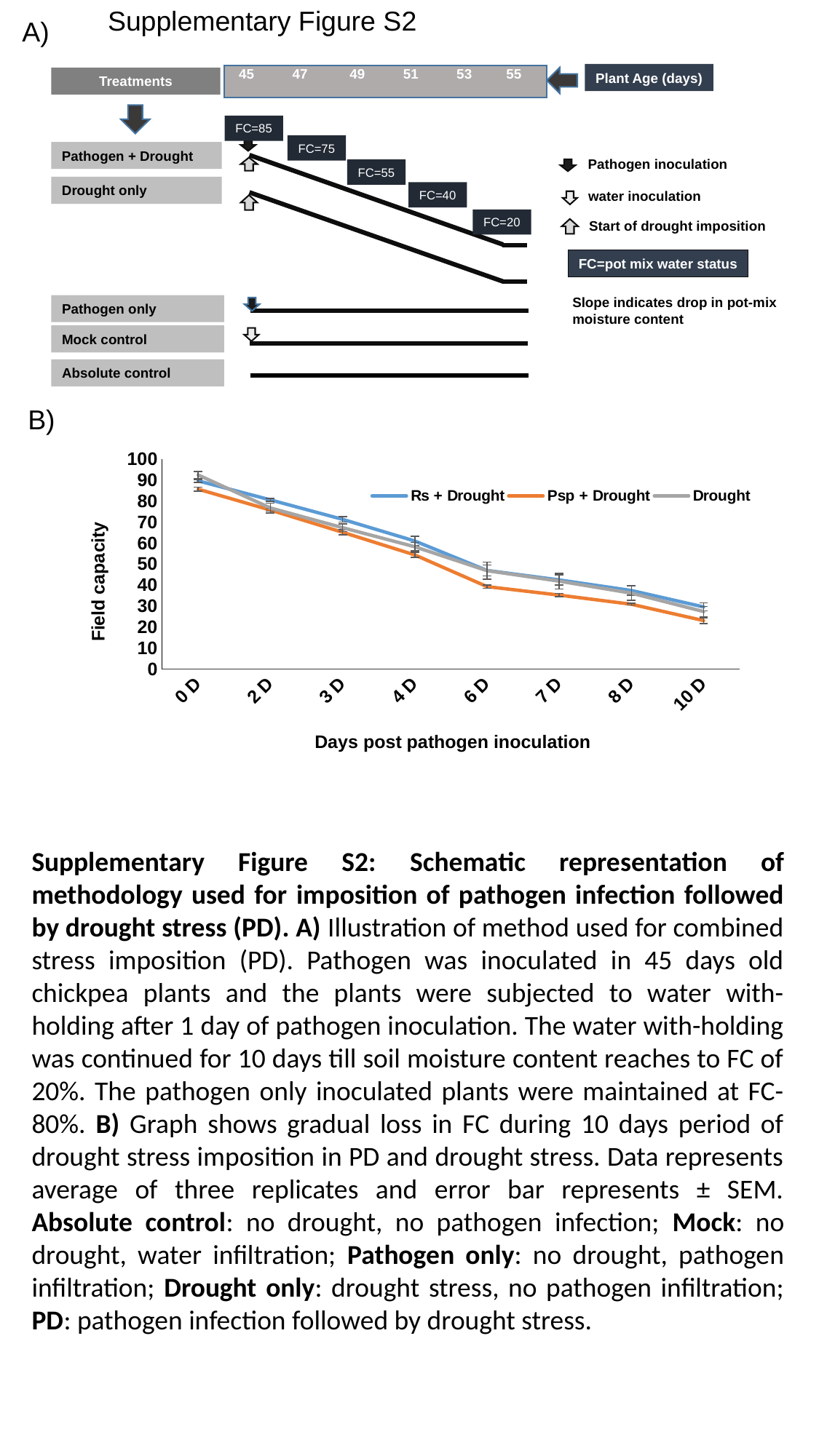

Supplementary Figure S2
A)
Plant Age (days)
 45 47 49 51 53 55
Treatments
Pathogen + Drought
Drought only
Pathogen only
Mock control
Absolute control
Pathogen inoculation
water inoculation
Start of drought imposition
FC=pot mix water status
Slope indicates drop in pot-mix moisture content
FC=85
FC=75
FC=55
FC=40
FC=20
B)
### Chart
| Category | Rs + Drought | Psp + Drought | Drought |
|---|---|---|---|
| 0 D | 89.72303206997083 | 85.73858114674441 | 92.51700680272108 |
| 2 D | 80.63654033041787 | 75.80174927113701 | 76.79786200194363 |
| 3 D | 71.37998056365403 | 65.20894071914479 | 67.3955296404276 |
| 4 D | 61.05442176870747 | 54.42176870748299 | 58.309037900874635 |
| 6 D | 47.03595724003887 | 39.33430515063168 | 46.938775510204074 |
| 7 D | 42.517006802721085 | 35.25267249757045 | 41.93391642371234 |
| 8 D | 37.46355685131195 | 30.903790087463552 | 36.224489795918366 |
| 10 D | 29.640427599611268 | 23.12925170068027 | 27.42954324586977 |Days post pathogen inoculation
Supplementary Figure S2: Schematic representation of methodology used for imposition of pathogen infection followed by drought stress (PD). A) Illustration of method used for combined stress imposition (PD). Pathogen was inoculated in 45 days old chickpea plants and the plants were subjected to water with-holding after 1 day of pathogen inoculation. The water with-holding was continued for 10 days till soil moisture content reaches to FC of 20%. The pathogen only inoculated plants were maintained at FC-80%. B) Graph shows gradual loss in FC during 10 days period of drought stress imposition in PD and drought stress. Data represents average of three replicates and error bar represents ± SEM. Absolute control: no drought, no pathogen infection; Mock: no drought, water infiltration; Pathogen only: no drought, pathogen infiltration; Drought only: drought stress, no pathogen infiltration; PD: pathogen infection followed by drought stress.
