# Supplementary material for: Understanding the Impact of Drought on Foliar and Xylem Invading Bacterial Pathogen Stress in Chickpea
Source: Front Plant Sci. 2016 Jun 21;7:902. doi: 10.3389/fpls.2016.00902 (PMC4914590; doi:10.3389/fpls.2016.00902)
Supplement: Supplementary file 5 [file Presentation3.PPTX]

## Slide 1
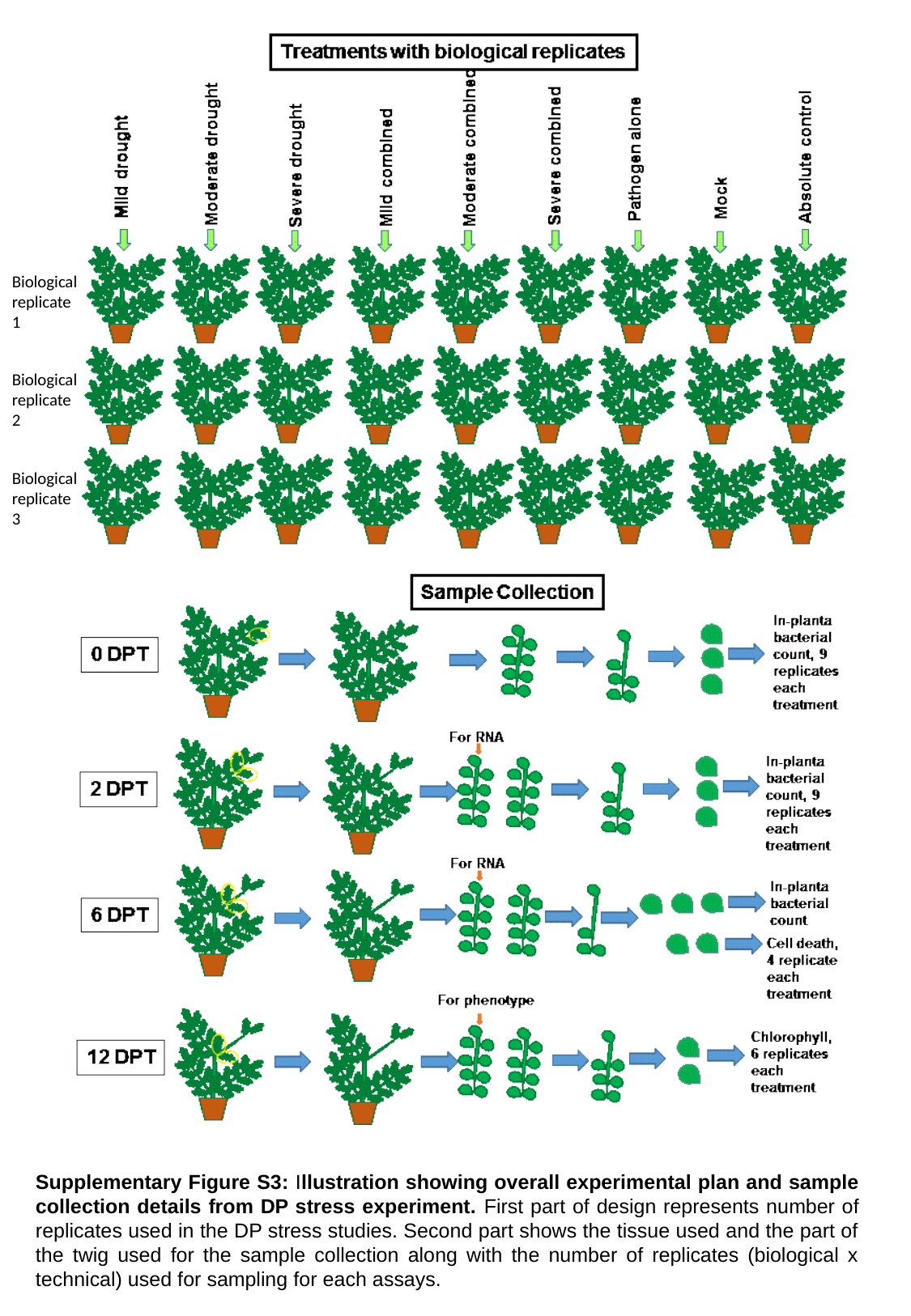

Biological replicate 1
Biological replicate 2
Biological replicate 3
Supplementary Figure S3: Illustration showing overall experimental plan and sample collection details from DP stress experiment. First part of design represents number of replicates used in the DP stress studies. Second part shows the tissue used and the part of the twig used for the sample collection along with the number of replicates (biological x technical) used for sampling for each assays.
