# Supplementary material for: Understanding the Impact of Drought on Foliar and Xylem Invading Bacterial Pathogen Stress in Chickpea
Source: Front Plant Sci. 2016 Jun 21;7:902. doi: 10.3389/fpls.2016.00902 (PMC4914590; doi:10.3389/fpls.2016.00902)
Supplement: Supplementary file 6 [file Presentation4.PPTX]

## Slide 1
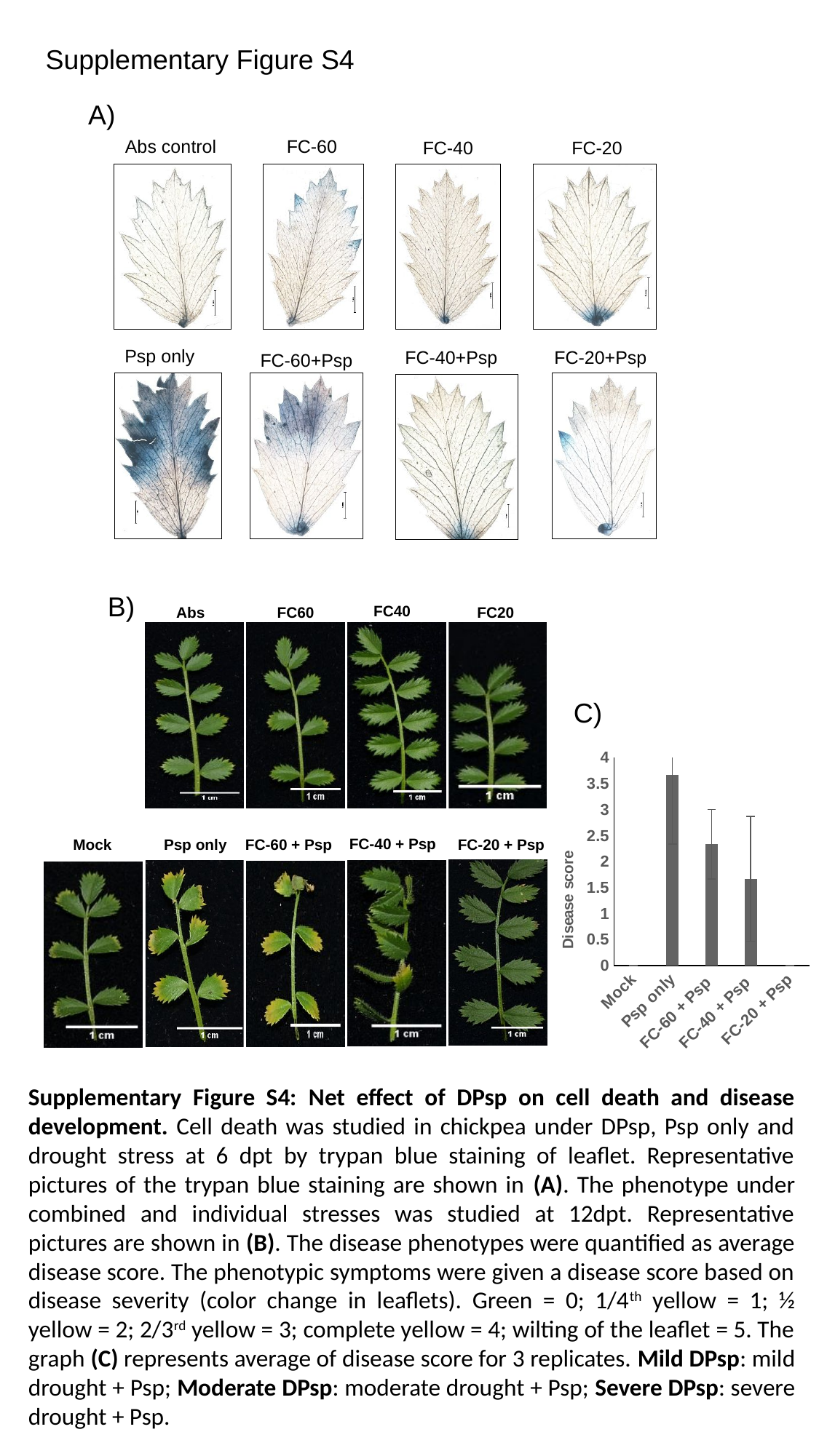

Supplementary Figure S4
A)
Abs control
FC-60
FC-40
FC-20
Psp only
FC-40+Psp
FC-20+Psp
FC-60+Psp
B)
FC40
FC20
Abs
FC60
FC-40 + Psp
Mock
FC-20 + Psp
Psp only
FC-60 + Psp
C)
### Chart
| Category | |
|---|---|
| Mock | 0.0 |
| Psp only | 3.6666666666666665 |
| FC-60 + Psp | 2.3333333333333335 |
| FC-40 + Psp | 1.6666666666666667 |
| FC-20 + Psp | 0.0 |Supplementary Figure S4: Net effect of DPsp on cell death and disease development. Cell death was studied in chickpea under DPsp, Psp only and drought stress at 6 dpt by trypan blue staining of leaflet. Representative pictures of the trypan blue staining are shown in (A). The phenotype under combined and individual stresses was studied at 12dpt. Representative pictures are shown in (B). The disease phenotypes were quantified as average disease score. The phenotypic symptoms were given a disease score based on disease severity (color change in leaflets). Green = 0; 1/4th yellow = 1; ½ yellow = 2; 2/3rd yellow = 3; complete yellow = 4; wilting of the leaflet = 5. The graph (C) represents average of disease score for 3 replicates. Mild DPsp: mild drought + Psp; Moderate DPsp: moderate drought + Psp; Severe DPsp: severe drought + Psp.
