# Supplementary material for: Understanding the Impact of Drought on Foliar and Xylem Invading Bacterial Pathogen Stress in Chickpea
Source: Front Plant Sci. 2016 Jun 21;7:902. doi: 10.3389/fpls.2016.00902 (PMC4914590; doi:10.3389/fpls.2016.00902)
Supplement: Supplementary file 7 [file Presentation5.PPTX]

## Slide 1
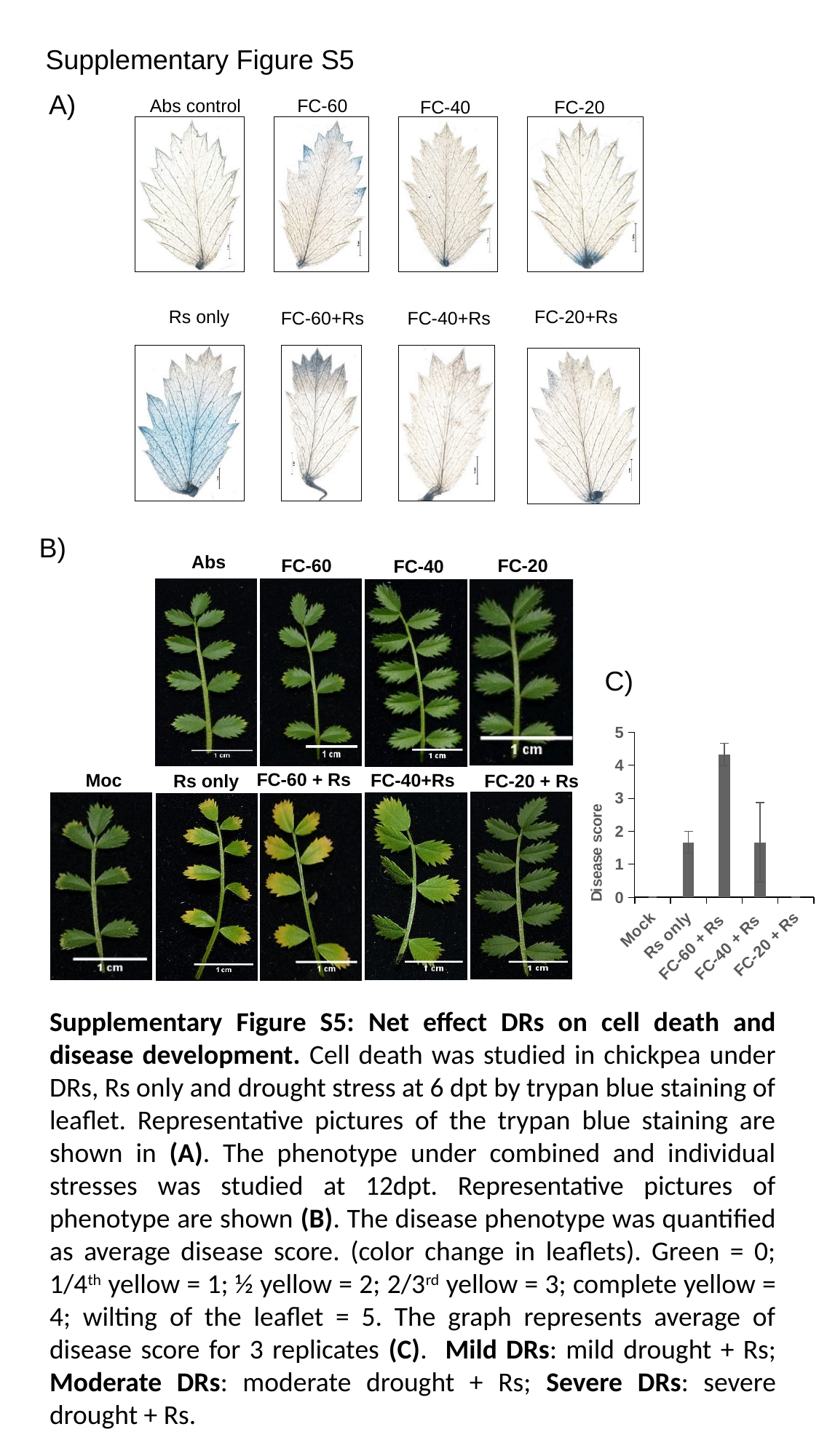

Supplementary Figure S5
A)
Abs control
FC-60
FC-40
FC-20
Rs only
FC-20+Rs
FC-60+Rs
FC-40+Rs
B)
Abs
FC-20
FC-60
FC-40
C)
### Chart
| Category | |
|---|---|
| Mock | 0.0 |
| Rs only | 1.6666666666666667 |
| FC-60 + Rs | 4.333333333333333 |
| FC-40 + Rs | 1.6666666666666667 |
| FC-20 + Rs | 0.0 |FC-60 + Rs
FC-40+Rs
Mock
Rs only
FC-20 + Rs
Supplementary Figure S5: Net effect DRs on cell death and disease development. Cell death was studied in chickpea under DRs, Rs only and drought stress at 6 dpt by trypan blue staining of leaflet. Representative pictures of the trypan blue staining are shown in (A). The phenotype under combined and individual stresses was studied at 12dpt. Representative pictures of phenotype are shown (B). The disease phenotype was quantified as average disease score. (color change in leaflets). Green = 0; 1/4th yellow = 1; ½ yellow = 2; 2/3rd yellow = 3; complete yellow = 4; wilting of the leaflet = 5. The graph represents average of disease score for 3 replicates (C). Mild DRs: mild drought + Rs; Moderate DRs: moderate drought + Rs; Severe DRs: severe drought + Rs.
