# Supplementary material for: Understanding the Impact of Drought on Foliar and Xylem Invading Bacterial Pathogen Stress in Chickpea
Source: Front Plant Sci. 2016 Jun 21;7:902. doi: 10.3389/fpls.2016.00902 (PMC4914590; doi:10.3389/fpls.2016.00902)
Supplement: Supplementary file 8 [file Presentation6.PPTX]

## Slide 1
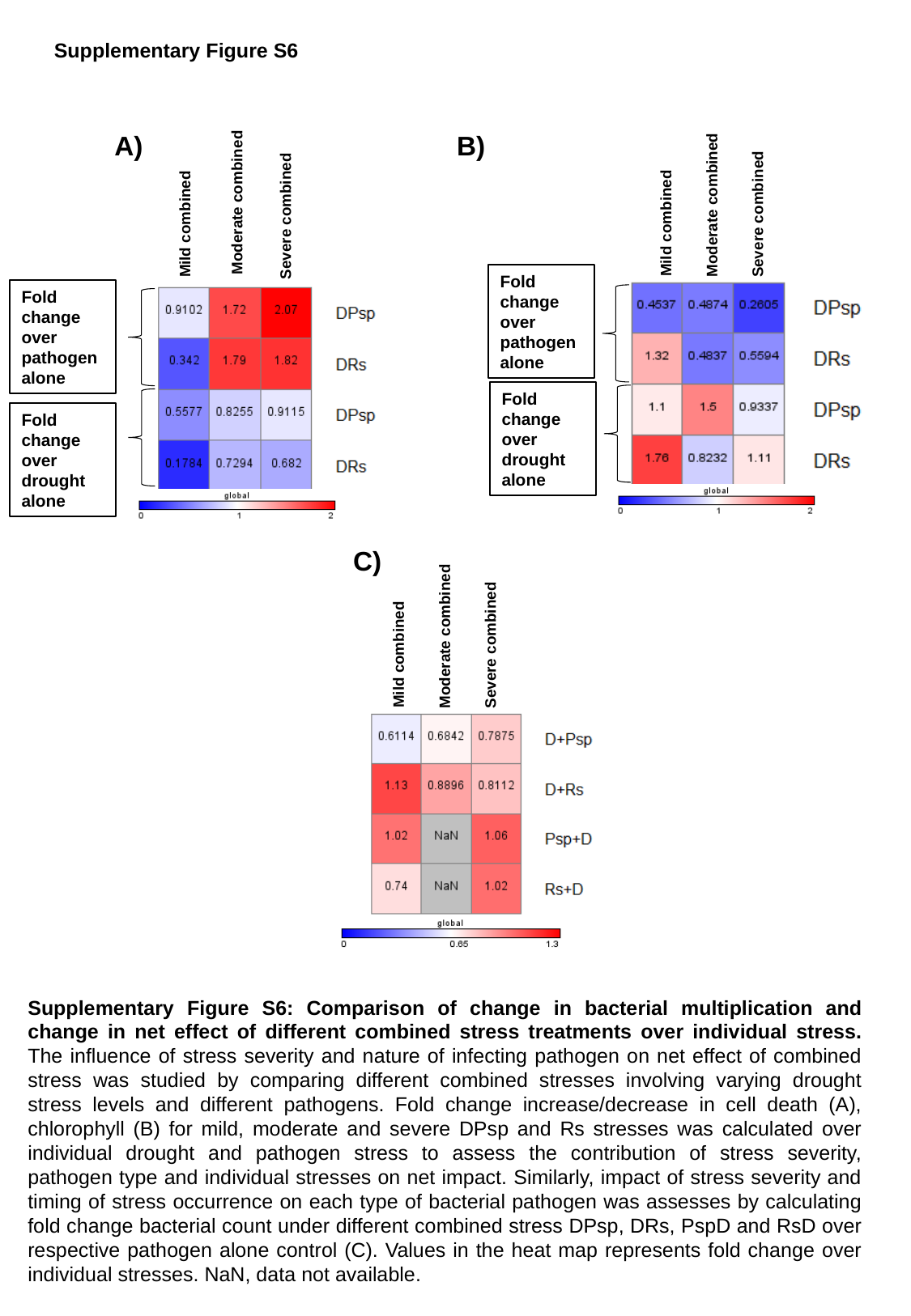

Supplementary Figure S6
Moderate combined
Severe combined
Mild combined
Moderate combined
Severe combined
Mild combined
A)
B)
Fold change over pathogen alone
Fold change over pathogen alone
Fold change over drought alone
Fold change over drought alone
C)
Moderate combined
Severe combined
Mild combined
Supplementary Figure S6: Comparison of change in bacterial multiplication and change in net effect of different combined stress treatments over individual stress. The influence of stress severity and nature of infecting pathogen on net effect of combined stress was studied by comparing different combined stresses involving varying drought stress levels and different pathogens. Fold change increase/decrease in cell death (A), chlorophyll (B) for mild, moderate and severe DPsp and Rs stresses was calculated over individual drought and pathogen stress to assess the contribution of stress severity, pathogen type and individual stresses on net impact. Similarly, impact of stress severity and timing of stress occurrence on each type of bacterial pathogen was assesses by calculating fold change bacterial count under different combined stress DPsp, DRs, PspD and RsD over respective pathogen alone control (C). Values in the heat map represents fold change over individual stresses. NaN, data not available.
