# Supplementary material for: Understanding the Impact of Drought on Foliar and Xylem Invading Bacterial Pathogen Stress in Chickpea
Source: Front Plant Sci. 2016 Jun 21;7:902. doi: 10.3389/fpls.2016.00902 (PMC4914590; doi:10.3389/fpls.2016.00902)
Supplement: Supplementary file 9 [file Presentation7.PPTX]

## Slide 1
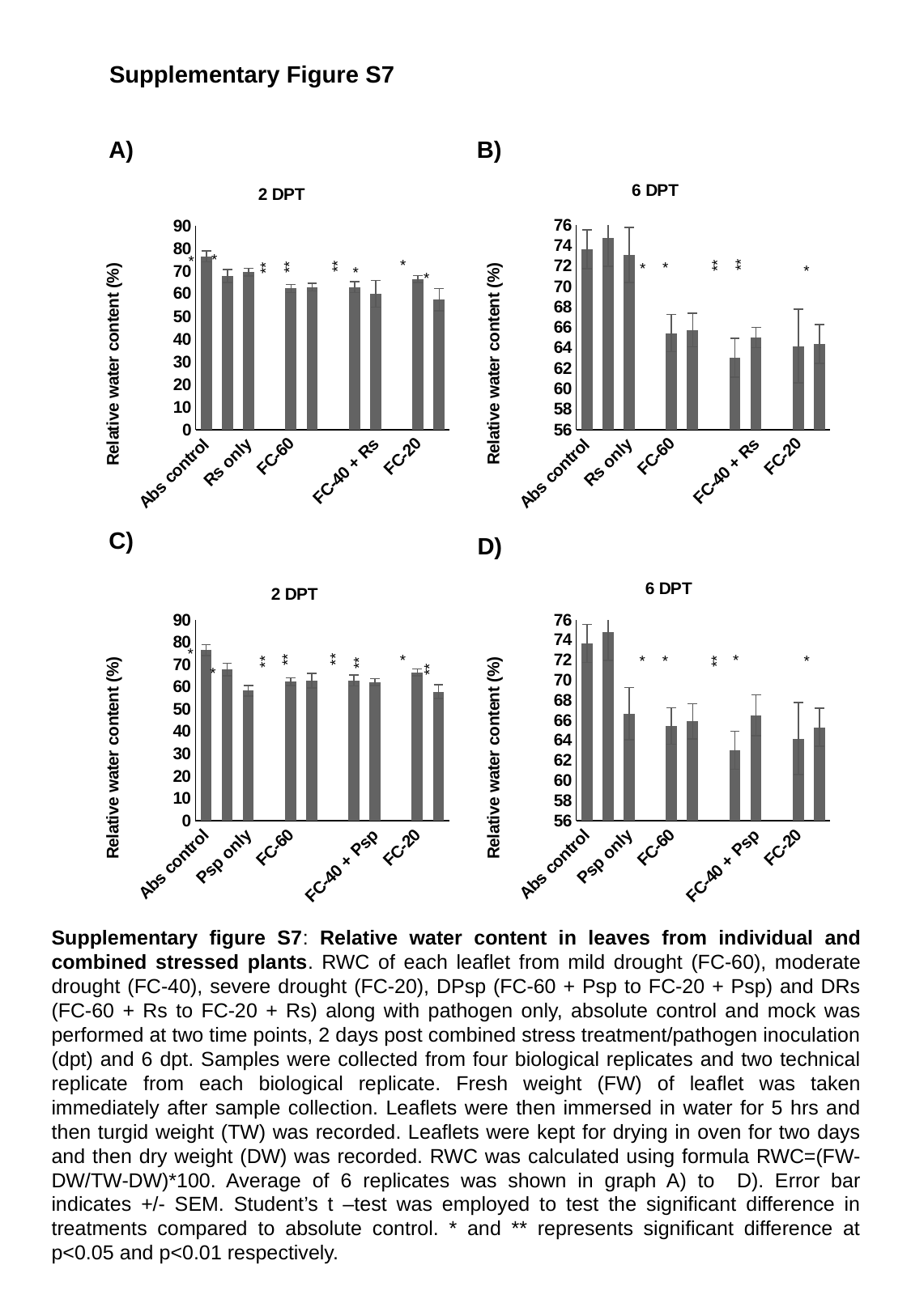

Supplementary Figure S7
A)
B)
### Chart: 6 DPT
| Category | |
|---|---|
| Abs control | 73.65668236405295 |
| Mock | 74.7617531961798 |
| Rs only | 73.10403010492445 |
| | None |
| FC-60 | 65.43962407668582 |
| FC-60 + Rs | 65.7419509733069 |
| | None |
| FC-40 | 63.026977849479316 |
| FC-40 + Rs | 65.02319709811405 |
| | None |
| FC-20 | 64.17870973138676 |
| FC-20 + Rs | 64.37737503662267 |*
*
*
**
**
### Chart: 2 DPT
| Category | |
|---|---|
| Abs control | 76.48953546976576 |
| Mock | 67.80499084571372 |
| Rs only | 69.55542282182094 |
| | None |
| FC-60 | 62.33535828896893 |
| FC-60 + Rs | 62.99073815033154 |
| | None |
| FC-40 | 62.94443680942158 |
| FC-40 + Rs | 59.95753503560794 |
| | None |
| FC-20 | 66.55836035033347 |
| FC-20 + Rs | 57.38906813759607 |*
*
*
**
**
**
*
*
C)
D)
### Chart: 2 DPT
| Category | |
|---|---|
| Abs control | 76.48953546976576 |
| Mock | 67.80499084571372 |
| Psp only | 58.30307558292096 |
| | None |
| FC-60 | 62.33535828896893 |
| FC-60 + Psp | 62.78262854499008 |
| | None |
| FC-40 | 62.94443680942158 |
| FC-40 + Psp | 62.187042877736516 |
| | None |
| FC-20 | 66.55836035033347 |
| FC-20 + Psp | 57.89003300506676 |*
*
*
**
**
**
**
**
### Chart: 6 DPT
| Category | |
|---|---|
| Abs control | 73.65668236405295 |
| Mock | 74.7617531961798 |
| Psp only | 66.67592714431957 |
| | None |
| FC-60 | 65.43962407668582 |
| FC-60 + Psp | 65.90698403542854 |
| | None |
| FC-40 | 63.026977849479316 |
| FC-40 + Psp | 66.50057120971263 |
| | None |
| FC-20 | 64.17870973138676 |
| FC-20 + Psp | 65.3129380912812 |*
*
*
*
**
Supplementary figure S7: Relative water content in leaves from individual and combined stressed plants. RWC of each leaflet from mild drought (FC-60), moderate drought (FC-40), severe drought (FC-20), DPsp (FC-60 + Psp to FC-20 + Psp) and DRs (FC-60 + Rs to FC-20 + Rs) along with pathogen only, absolute control and mock was performed at two time points, 2 days post combined stress treatment/pathogen inoculation (dpt) and 6 dpt. Samples were collected from four biological replicates and two technical replicate from each biological replicate. Fresh weight (FW) of leaflet was taken immediately after sample collection. Leaflets were then immersed in water for 5 hrs and then turgid weight (TW) was recorded. Leaflets were kept for drying in oven for two days and then dry weight (DW) was recorded. RWC was calculated using formula RWC=(FW-DW/TW-DW)*100. Average of 6 replicates was shown in graph A) to D). Error bar indicates +/- SEM. Student’s t –test was employed to test the significant difference in treatments compared to absolute control. * and ** represents significant difference at p<0.05 and p<0.01 respectively.
