# Supplementary material for: Understanding the Impact of Drought on Foliar and Xylem Invading Bacterial Pathogen Stress in Chickpea
Source: Front Plant Sci. 2016 Jun 21;7:902. doi: 10.3389/fpls.2016.00902 (PMC4914590; doi:10.3389/fpls.2016.00902)
Supplement: Supplementary file 10 [file Presentation8.PPTX]

## Slide 1
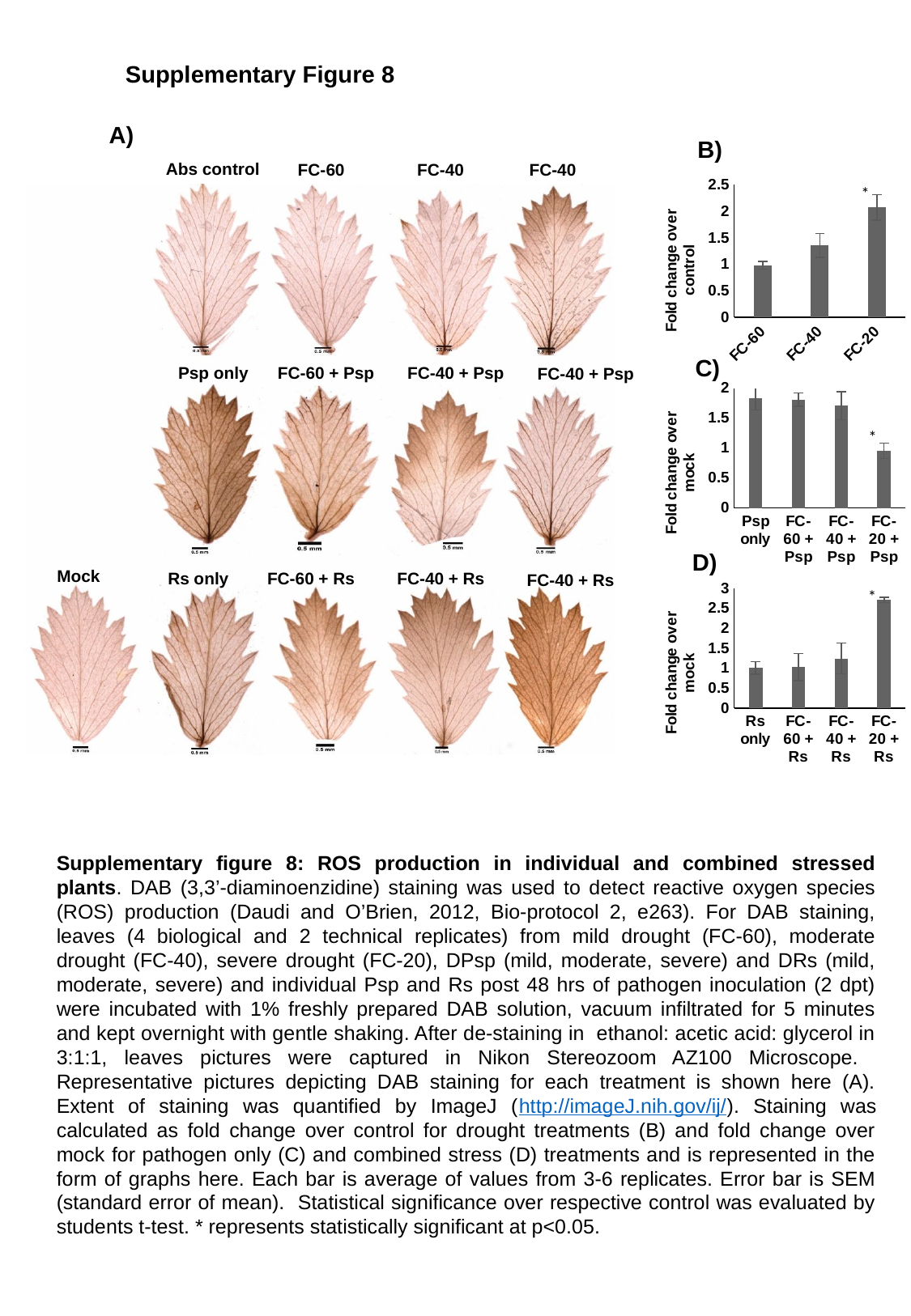

Supplementary Figure 8
A)
B)
Abs control
FC-40
FC-40
FC-60
Psp only
FC-60 + Psp
FC-40 + Psp
FC-40 + Psp
Mock
Rs only
FC-60 + Rs
FC-40 + Rs
FC-40 + Rs
### Chart
| Category | |
|---|---|
| FC-60 | 0.9826830431473323 |
| FC-40 | 1.3560971751362043 |
| FC-20 | 2.0692581745077843 |*
C)
### Chart
| Category | |
|---|---|
| Psp only | 1.8319280951325283 |
| FC-60 + Psp | 1.8070460408616469 |
| FC-40 + Psp | 1.7072137097969777 |
| FC-20 + Psp | 0.95371387002028 |*
D)
### Chart
| Category | |
|---|---|
| Rs only | 1.0037040286444106 |
| FC-60 + Rs | 1.0283882525691044 |
| FC-40 + Rs | 1.2398877859533821 |
| FC-20 + Rs | 2.707696584693838 |*
Supplementary figure 8: ROS production in individual and combined stressed plants. DAB (3,3’-diaminoenzidine) staining was used to detect reactive oxygen species (ROS) production (Daudi and O’Brien, 2012, Bio-protocol 2, e263). For DAB staining, leaves (4 biological and 2 technical replicates) from mild drought (FC-60), moderate drought (FC-40), severe drought (FC-20), DPsp (mild, moderate, severe) and DRs (mild, moderate, severe) and individual Psp and Rs post 48 hrs of pathogen inoculation (2 dpt) were incubated with 1% freshly prepared DAB solution, vacuum infiltrated for 5 minutes and kept overnight with gentle shaking. After de-staining in ethanol: acetic acid: glycerol in 3:1:1, leaves pictures were captured in Nikon Stereozoom AZ100 Microscope. Representative pictures depicting DAB staining for each treatment is shown here (A). Extent of staining was quantified by ImageJ (http://imageJ.nih.gov/ij/). Staining was calculated as fold change over control for drought treatments (B) and fold change over mock for pathogen only (C) and combined stress (D) treatments and is represented in the form of graphs here. Each bar is average of values from 3-6 replicates. Error bar is SEM (standard error of mean). Statistical significance over respective control was evaluated by students t-test. * represents statistically significant at p<0.05.
