# Supplementary material for: Understanding the Impact of Drought on Foliar and Xylem Invading Bacterial Pathogen Stress in Chickpea
Source: Front Plant Sci. 2016 Jun 21;7:902. doi: 10.3389/fpls.2016.00902 (PMC4914590; doi:10.3389/fpls.2016.00902)
Supplement: Supplementary file 11 [file Presentation9.PPTX]

## Slide 1
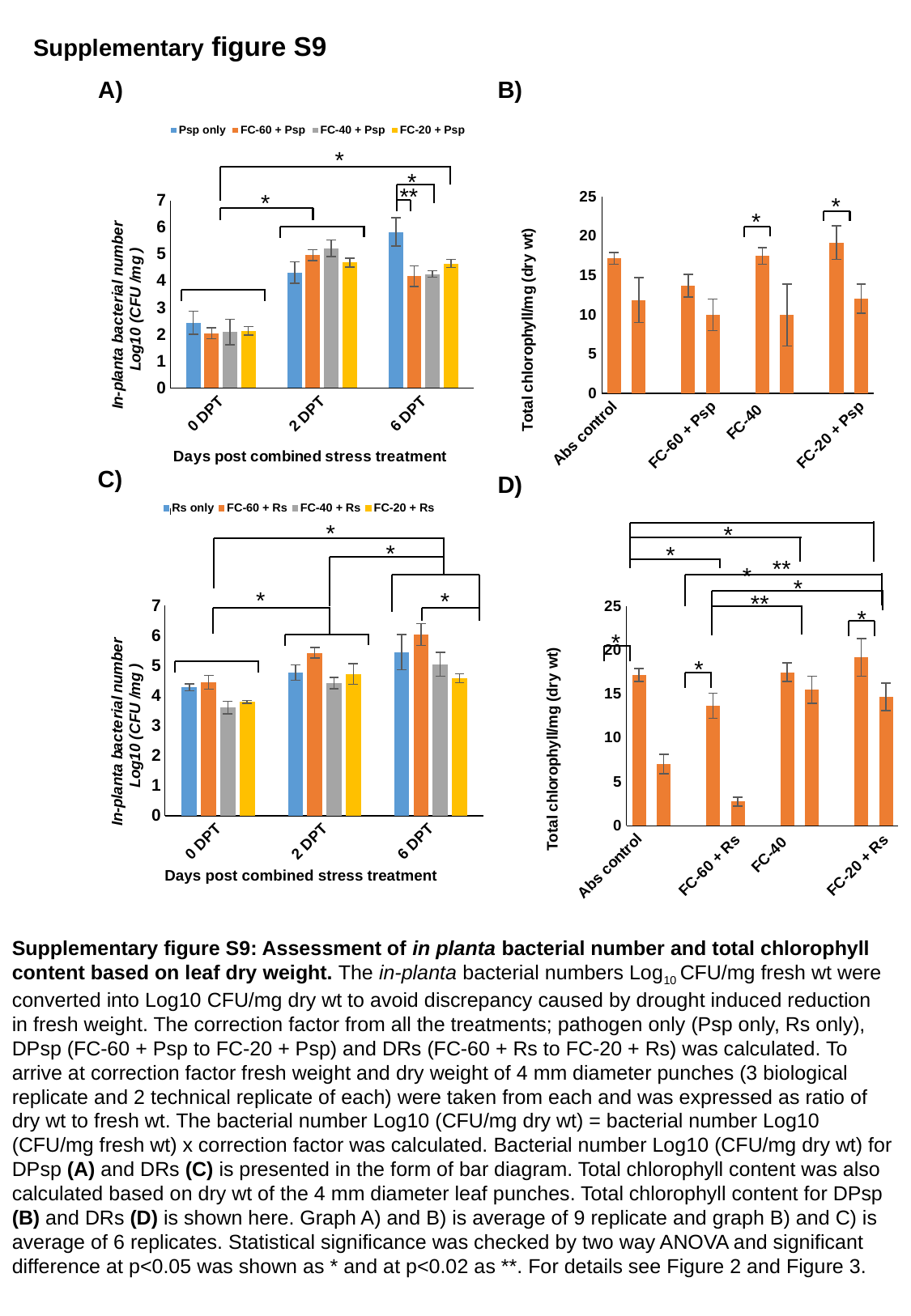

Supplementary figure S9
A)
B)
*
*
**
### Chart
| Category | Psp only | FC-60 + Psp | FC-40 + Psp | FC-20 + Psp |
|---|---|---|---|---|
| 0 DPT | 2.4272909922371615 | 2.0383744202382315 | 2.0872500842870307 | 2.1279456643766177 |
| 2 DPT | 4.306618972612586 | 4.953818818611045 | 5.217863184884855 | 4.679428183804004 |
| 6 DPT | 5.822410079831237 | 4.171790182630589 | 4.252775638019289 | 4.645551239765242 |*
### Chart
| Category | |
|---|---|
| Abs control | 17.141183333333334 |
| Psp only | 11.856187499999999 |
| | None |
| FC-60 | 13.65790196078431 |
| FC-60 + Psp | 9.961057692307692 |
| | None |
| FC-40 | 17.462588235294113 |
| FC-40 + Psp | 9.944112903225804 |
| | None |
| FC-20 | 19.151986486486486 |
| FC-20 + Psp | 12.036055555555555 |*
*
C)
D)
*
*
*
*
### Chart
| Category | Rs only | FC-60 + Rs | FC-40 + Rs | FC-20 + Rs |
|---|---|---|---|---|
| 0 DPT | 4.266140806429109 | 4.439177279003527 | 3.5964797863215767 | 3.7850188038668904 |
| 2 DPT | 4.761407220493353 | 5.4209863955287405 | 4.414242584601941 | 4.713331868664626 |
| 6 DPT | 5.441741991696474 | 6.031675996341274 | 5.036948641271451 | 4.572826238773982 |Days post combined stress treatment
*
*
**
*
**
### Chart
| Category | |
|---|---|
| Abs control | 17.141183333333334 |
| Rs only | 7.019270531400967 |
| | None |
| FC-60 | 13.65790196078431 |
| FC-60 + Rs | 2.7608777777777775 |
| | None |
| FC-40 | 17.462588235294113 |
| FC-40 + Rs | 15.466851190476191 |
| | None |
| FC-20 | 19.151986486486486 |
| FC-20 + Rs | 14.665091666666667 |*
*
*
*
Supplementary figure S9: Assessment of in planta bacterial number and total chlorophyll content based on leaf dry weight. The in-planta bacterial numbers Log10 CFU/mg fresh wt were converted into Log10 CFU/mg dry wt to avoid discrepancy caused by drought induced reduction in fresh weight. The correction factor from all the treatments; pathogen only (Psp only, Rs only), DPsp (FC-60 + Psp to FC-20 + Psp) and DRs (FC-60 + Rs to FC-20 + Rs) was calculated. To arrive at correction factor fresh weight and dry weight of 4 mm diameter punches (3 biological replicate and 2 technical replicate of each) were taken from each and was expressed as ratio of dry wt to fresh wt. The bacterial number Log10 (CFU/mg dry wt) = bacterial number Log10 (CFU/mg fresh wt) x correction factor was calculated. Bacterial number Log10 (CFU/mg dry wt) for DPsp (A) and DRs (C) is presented in the form of bar diagram. Total chlorophyll content was also calculated based on dry wt of the 4 mm diameter leaf punches. Total chlorophyll content for DPsp (B) and DRs (D) is shown here. Graph A) and B) is average of 9 replicate and graph B) and C) is average of 6 replicates. Statistical significance was checked by two way ANOVA and significant difference at p<0.05 was shown as * and at p<0.02 as **. For details see Figure 2 and Figure 3.
